# Reactions of Lanthanide Ions with Glycolic Acid or Tartaric Acid in the Presence of Spermine: Potentiometric and Spectroscopic Studies

**DOI:** 10.3390/ijms26104477

**Published:** 2025-05-08

**Authors:** Michał Zabiszak, Justyna Frymark, Monika Skrobanska, Malgorzata T. Kaczmarek, Renata Jastrzab

**Affiliations:** Faculty of Chemistry, Adam Mickiewicz University in Poznan, Uniwersytetu Poznańskiego 8, 61-614 Poznan, Poland; justyna.frymark@amu.edu.pl (J.F.); monskr@amu.edu.pl (M.S.); gosiat@amu.edu.pl (M.T.K.); renatad@amu.edu.pl (R.J.)

**Keywords:** lanthanide ions, glycolic acid, tartaric acid, spermine, potentiometric, spectroscopy, coordination chemistry

## Abstract

In order to determine how lanthanide ions (La, Nd, Eu, Gd, Ho, Tb, Lu) coordinate in ternary systems with alpha-hydroxy acids (glycolic acid—GA; or tartaric acid—Tar) and spermine (Spm), potentiometric studies were conducted. In addition, binary systems of metal ions with alpha-hydroxy acids were studied, as well as the possibility of non-covalent interactions between the studied ligands. This study was carried out in aqueous solutions, and the composition of the formed complexes was confirmed using computer data analysis. Using spectroscopic measurements, the inner and outer coordination spheres of the central metal ion were determined. In ternary systems, via luminescent studies, the occurrence of the so-called ‘antenna effect’ was confirmed. These studies confirmed the formation of ternary complexes in which spermine can exist in the outer coordination sphere of lanthanide ions interacting non-covalently with an alpha-hydroxy acid in the inner coordination sphere.

## 1. Introduction

Lanthanide(III) ions exhibit luminescent properties, and their characteristic emission lines can be enhanced or quenched by the presence of ligands in their coordination sphere [1,2]. Their complex compounds have found medical applications in cancer diagnosis and the monitoring of the treatment process [3,4,5,6]. Due to their paramagnetic properties, lanthanide(III) ions and their complex compounds are widely used in magnetic resonance imaging (MRI). Gadolinium(III) ion complexes have found the widest application in this field [7]. In addition to medical applications, these elements are also key in industry, where they play an important role, among other things, in the production of strong magnets, which are used in electronics, oil refining and renewable energy technologies [8,9,10,11]. According to the assumptions of the theory of Hard and Soft Acids and Bases (HSAB), lanthanide(III) ions most prefer to form chemical compounds with ligands containing donor oxygen atoms, followed by ligands containing donor nitrogen or sulfur atoms. Ligands that contain donor oxygen atoms include α-hydroxy acids such as tartaric acid and glycolic acid. They are potential ligands and can form coordination complexes of mononuclear, binuclear and polymeric types [12,13,14].

α-hydroxy acids, due to their broad spectrum of action, have found their way into the cosmetics industry, where they mainly act as chemical exfoliants as an additive to cosmetics. Glycolic acid, due to being the smallest molecule among the α-hydroxy acids, easily penetrates the stratum corneum and has found the greatest cosmetic use [15,16,17]. α-hydroxy acids and their complexes are used not only in cosmetics but also in medicine: in digestive diseases, tartaric acid is used as a drug [18,19]. Furthermore, glycolic acid is used together with lactic acid to produce a Poly Lactic-Co-Glycolic Acid (PLGA) copolymer, used in the production of surgical threads, drugs, smart scaffolds for tissue engineering and biodegradable textiles [20]. Due to their structure, α-hydroxy acids, which contain a hydroxyl group and a minimum of one carboxylic group, exhibit the properties of both alcohols and carboxylic acids. The presence of donor oxygen atoms in α-hydroxy acids makes them potential ligands for metal ions such as lanthanide ions.

Biogenic polyamines exhibit different chemical properties to α-hydroxy acids, including spermine. Spermine has only amino groups in its structure, which causes it to exhibit alkaline properties. Spermine occurs naturally in living organisms, and its concentration level depends on the type of tissue and, most importantly, the age of the organism. The highest concentration of spermine was detected in the pancreas (4 mM) [21]. However, high levels of spermine have also been detected in the lung, liver, kidney, spleen and semen [22,23]. It is noteworthy that increased concentrations of polyamines have been observed in cancer cells [24]. Polyamines are associated with their strong basic character. These substances have a high affinity for the proton, which increases with the elongation of the methylene chain of the amine. Biogenic amines are completely protonated at pH 7.0 (physiological conditions) and are capable of forming non-covalent interactions with negatively charged donor groups, such as the carboxyl groups of α-hydroxy acids [12,25,26,27].

## 2. Results and Discussion

### 2.1. Equilibrium Study

**Figure 1:**
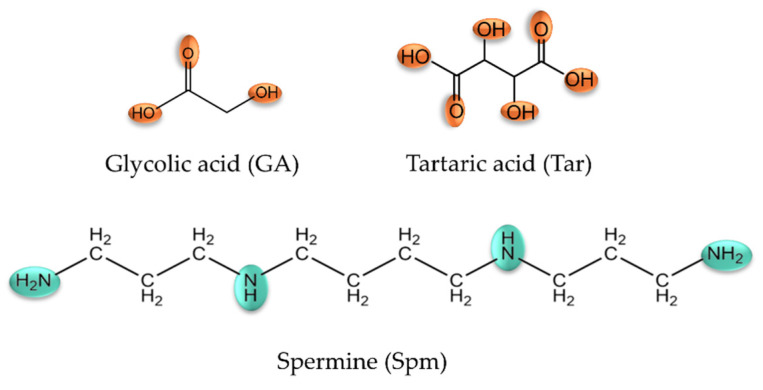
Structural formula of the studied ligands.

The first stage of the research work involved determining the protonation constants of naturally occurring acids (glycolic acid—GA; tartaric acid—Tar) and spermine (Spm). The results obtained on tartaric acid protonation and spermine were previously published in a paper on its complexation reaction with d-electron elements [28,29]. The determined value of the protonation constant of glycolic acid is 3.09. In the investigated pH range, the alpha-hydroxy acids’ donor groups (simultaneously sites of protonation) are the carboxyl groups. The value of the protonation constant of the hydroxyl group for alpha-hydroxy acids was determined using 13C NMR spectroscopy and ranges from approximately 11.6 to approximately 14.0 [30,31]. The hydroxy group can participate in coordination to lanthanide ions via its lone-pair electrons, and such coordination can occur with or without deprotonation, depending on the specific metal–ligand interaction and solution conditions; therefore, despite its high pKa, this group cannot be considered negligible in complexation studies within the pH range of 2.5–11.0. The relatively low values of the overall protonation constants of the acids studied suggest the deprotonation of carboxyl groups at low pH values, causing these compounds to exist in a completely deprotonated form over a wide range of the studied pH range, facilitating the complexation of metal ions. The patterns of the ligands studied, with potential coordination sites marked, are shown in Figure 1.

#### 2.1.1. Binary Systems of Lanthanide(III) Ion/α-Hydroxyacids

The binary systems of tartaric acid with lanthanide(III) ions were described in a previous study [32], and the obtained results were used for a computer analysis of the data obtained from potentiometric titrations of ternary systems. The study of the Ln(III)/GA system was conducted in the pH range of 2.5 to 11.0. The results of the potentiometric measurements and the computer analysis of the data are presented in Table 1. The computer analysis of the potentiometric data included the hydrolysis constants of lanthanide(III) ions [32].

Based on the potentiometric titration data obtained, computer-generated form distribution curves for the binary systems were generated taking into account the protonation constants, hydrolysis constants and stability constants of the complex compounds formed. In an analogous way, distribution diagrams were prepared for the studied metal ion-free system and ternary systems (stability constants of binary complexes were included). Complexation reactions in glycolic acid systems with lanthanide(III) ions start at a pH around 5.0 by forming a monohydroxyl complex, while for the La(III)/GA system, only the dihydroxylated form of LaGA(OH)_2_ is observed. The convergence of the theoretical curve with the experimental curve, along with a comparison to the theoretical curve that does not account for complex formation, confirms the validity of the proposed model.

**Figure 2:**
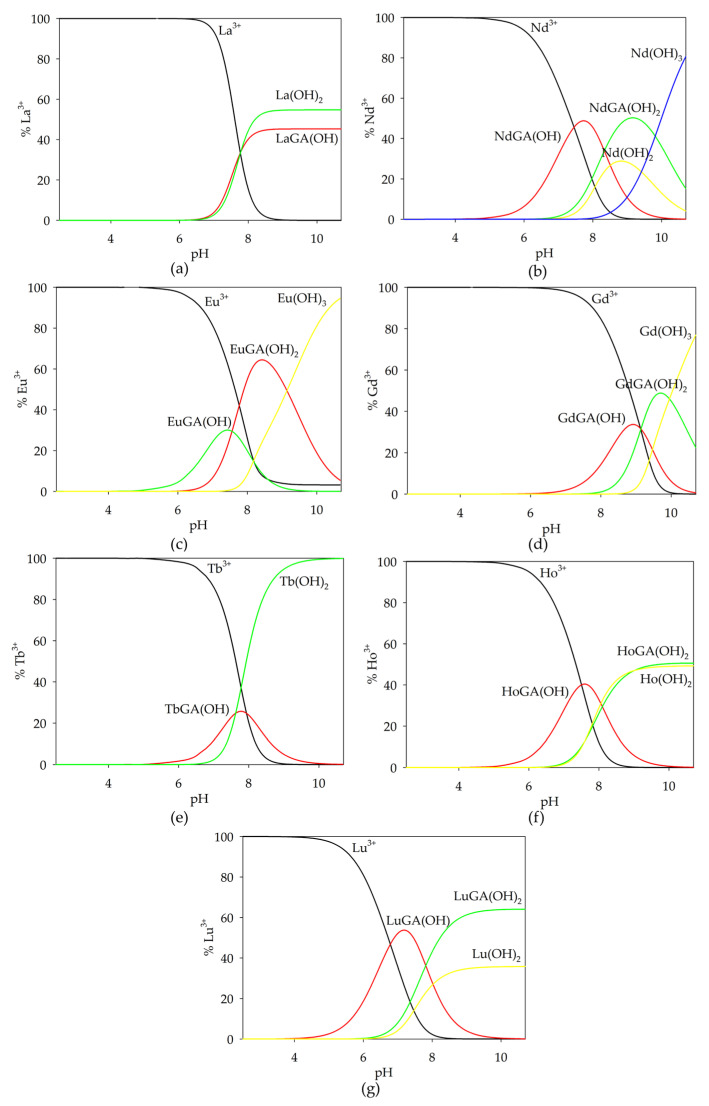
Distribution diagrams for the following systems: (**a**) La(III)/GA; (**b**) Nd(III)/GA; (**c**) Eu(III)/GA; (**d**) Gd(III)/GA; (**e**) Tb(III)/GA; (**f**) Ho(III)/GA; (**g**) Lu(III)/GA. c_metal ion_ = 1 × 10^−3^ mol dm^−3^; c_GA_ = 1 × 10^−3^ mol dm^−3^.

Monohydroxyl complexes reach their maximum concentration at a pH of around 7.5, which coordinates (depending on the lanthanide ions present in the system) 30–50% of the metal ions (Figure 2). The LnGA(OH)_2_ form is formed in the system at a pH around 6.0 and competes with lanthanide hydroxides, binding a maximum of 60% of the lanthanide(III) ions. For the Tb(III)/GA system, only the TbGA(OH) form was observed, which forms in a low concentration. It is noteworthy that for the gadolinium(III) ion system, the formed complex compounds exhibit a higher alkalinity compared to the analogous forms in the other investigated systems. Furthermore, the stability constants of the analogous forms in the systems increase with the increase in nuclear charge from lanthanum to lutetium. This phenomenon is called “lanthanide contraction”.

An analysis of the distribution curves of the complexes in the investigated systems indicates a relatively small participation of lanthanide ions in the complexation process with glycolic acid. The low concentrations of complex forms likely result from their formation at high pH values when lanthanide hydroxides are present in the systems, which occur as poorly soluble precipitates.

#### 2.1.2. Binary Systems of α-Hydroxyacids/Spermine

Considering the protonation range of the ligands studied, there are conditions that allow the formation of adducts, where the potential positive interaction center contains the protonated nitrogen atoms of polyamines, while the negative center contains the oxygen atoms of the carboxylic groups of the α-hydroxyacids. As a result of the non-covalent interactions of polyamines with α-hydroxyacids in binary systems (without a metal ion), the acid–base character of the ligands is changed. Taking advantage of the fact that the formation of such molecular complexes is accompanied by the release of protons, a potentiometric method was used to observe these processes.

Hx(Spm) + Hy(AHA) ⇆ (Spm)H(x + y − n)(AHA) +nH+ (AHA − α-hydroxy acid)

**Figure 3:**
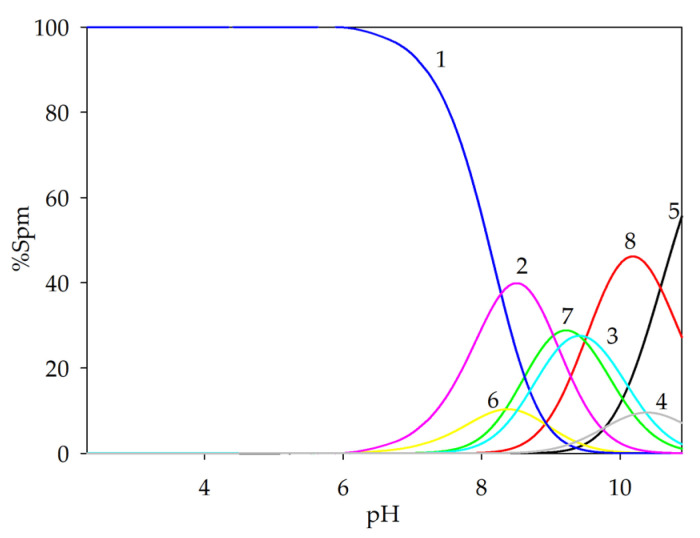
Distribution diagrams for the GA/Spm system; (1—H_4_Spm; 2—H_3_Spm; 3—H_2_Spm; 4—HSpm; 5—Spm; 6—(GA)(H_3_Spm); 7—(GA)(H_2_Spm); 8—(GA)(HSpm); c_GA_ = 1 × 10^−3^ mol dm^−3^; c_Spm_ = 1 × 10^−3^ mol dm^−3^.

The non-covalent interactions of spermine with L-tartaric acid were described in our previous work [32]. Three forms were found to be formed in the GA/Spm system: (GA)(H_3_Spm), (GA)(H_2_Spm) and (GA)(HSpm). Their overall stability constants (log*β*) are 33.09 (3), 24.62 (2) and 15.11 (1). The equilibrium constants of the formation reaction (log*K_e_*) are, respectively, the following: 2.70; 3.34; 4.20. An analysis of these values indicates the role of the structural factor in the importance of spatial affinity during the reaction of spermine with glycolic acid. The spermine complexation reaction with glycolic acid begins at a pH of approximately 6.5 with the formation of the (GA)(H_3_Spm) adduct, which disappears at pH = 10.0 (Figure 3). This form binds the maximum amount of ligands (15%) at pH = 8.0. The diproton complex at its maximum concentration binds about 30% of the ligands present in the solution, and at pH 9.0, deprotonated glycolic acid reacts with partially deprotonated polyamine (HL).

#### 2.1.3. Ternary Systems of Ln(III)/Tar/Spm

The investigation of the Ln(III)/Tar/Spm system was carried out in the pH range of 2.5–11.0, revealing the formation of only protonated forms of the type Ln(H_x_Tar)(H_y_Spm) (where x = 1 or 0; y = 1–4). The calculations included the protonation constants of the ligands, the hydrolysis constants of the metal ions and the stability constants of the binary systems (Ln(III)/Tar [31]; Ln(III)/Spm [29]; Tar/Spm [33]). In the Gd(III)/Tar/Spm system, no formation of heteroligand complexes was observed, as confirmed by the overlap of experimental titration curves with those simulated computationally, taking into account only binary complexes and low-complex ligands. The absence of ternary forms in the gadolinium-ion system was likely due to the low overall stability constants of the complexes, preventing their identification, caused by the so-called “gadolinium break”. The results of the potentiometric studies and the computer data analysis are presented in Table 2.

**Figure 4:**
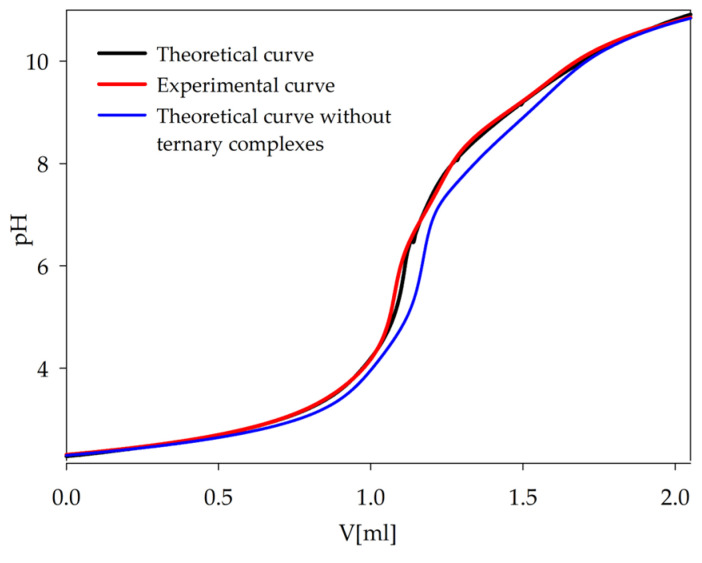
Comparison of theoretical curves with experimental curve of Ln(III)/Tar/Spm system; c_Ln_ = 1 × 10^−3^ mol dm^−3^; c_Tar_ = 1 × 10^−3^ mol dm^−3^; c_Spm_= 1 × 10^−3^ mol dm^−3^.

The occurrence of complexation reactions in ternary systems was confirmed by comparing the experimental potentiometric titration curve with the theoretical curves generated computationally using the HYPERQUAD2008 program (Figure 4). The deviation of the theoretical curve, which does not account for the formation of ternary complexes, further supports the validity of the proposed complex forms of lanthanide(III) ions in the investigated systems with a biogenic polyamine and a naturally occurring acid.

**Figure 5:**
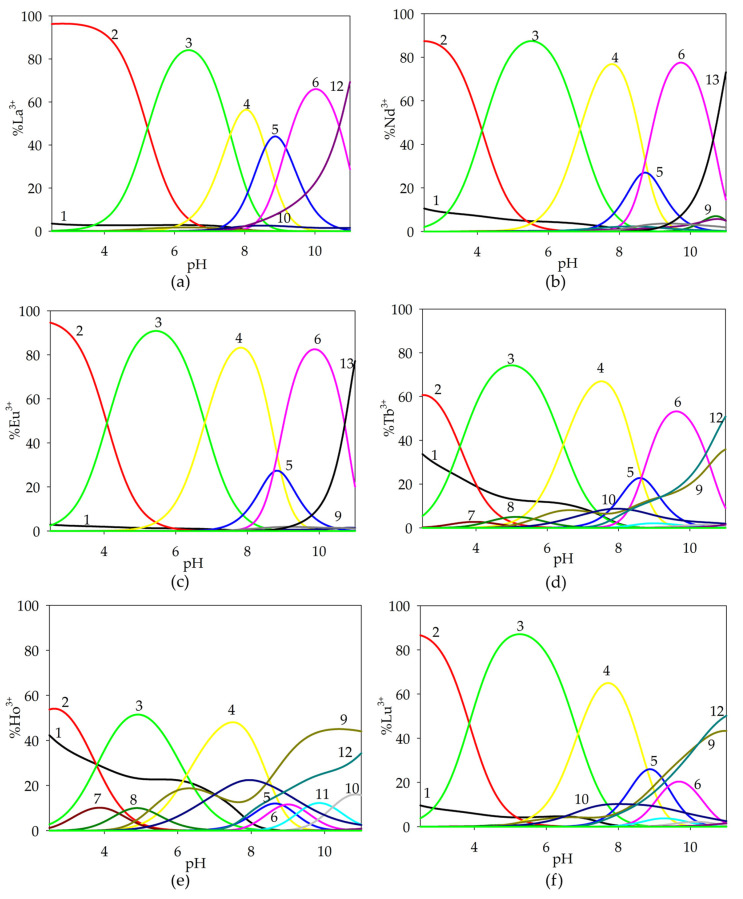
Distribution diagram for the studied systems: (**a**) La(III)/Tar/Spm; (**b**) Nd(III)/Tar/Spm; (**c**) Eu(III)/Tar/Spm; (**d**) Tb(III)/Tar/Spm; (**e**) Ho(III)/Tar/Spm; (**f**) Lu(III)/Tar/Spm (1—Ln^3+^; 2—Ln(HTar)(H_4_Spm); 3—Ln(Tar)(H_4_Spm); 4—Ln(Tar)(H_3_Spm); 5—Ln(Tar)(H_2_Spm); 6—Ln(Tar)(HSpm); 7—Ln(HTar)(Tar); 8—LnTar_2_; 9—LnTar_2_(OH); 10—LnTar(OH); 11–LnSpm; 12—Ln(OH)_2_; 13—Ln(OH)_3_). c_metal ion_ = 1 × 10^−3^ mol dm^−3^; c_Tar_ = 1 × 10^−3^ mol dm^−3^; c_Spm_= 1 × 10^−3^ mol dm^−3^.

The complexation reactions were conducted under conditions where the lanthanide ions were already observed to form Ln(HTar)(H_4_Spm) species, binding from 50% to 95% of the free metal ions present in the solution at pH 2.5. Within the dominance range of this complex, the Ln(Tar)(H_4_Spm) form was formed, which in the pH range of 4.0–7.0 was dominant, reaching its maximum concentration at around pH 5.0 (Figure 5). The involvement of fully protonated spermine interacting non-covalently with tartaric acid within the inner coordination sphere of the central atom was observed.

From around pH 5.0, the formation of the Ln(Tar)(H_3_Spm) complex began in the system, and occurred until pH 9.5, with the maximum concentration of this form occurring at pH 8.0. It should be noted that the acid–base properties of spermine changed, causing the earlier deprotonation of the amino group and its coordination with the metal ion.

The diprotonated complex Ln(Tar)(H_2_Spm) was observed in the dominance range of the Ln(Tar)(H_3_Spm) and monoprotonated Ln(Tar)(HSpm) complexes, reaching its maximum concentration (approximately 20%) at around pH 9.0. Only for the La(III)/Tar/Spm system did this complex predominate, coordinating 40% of the lanthanum(III) ions.

At pH 8.0, the formation of the Ln(Tar)(HSpm) form began. This complex occurred in the solution up to pH 11.0, and it bound the maximum amount of Ln(III) ions around pH 10.0. The composition of the complex suggests the involvement of three nitrogen atoms in inner coordination sphere with the lanthanide(III) ion and the interaction between the protonated amino group and the carboxyl group of the tartaric acid.

It is noteworthy that for ternary systems containing terbium(III), holmium(III) and lutetium(III) ions, the occurrence of double complexes of Ln(III)/Spm and Ln(III)/Tar was observed in the investigated pH range. At higher pH values, these complexes were the dominant forms in the Ho(III)/Spm/Tar and Lu(III)/Spm/Tar systems. The presence of double complexes for lanthanide ions characterized by a greater atomic mass confirms the tendency of rare earth element ions to increase the stability of complexes in the series from lanthanum to lutetium.

An analysis of equilibrium constants for the proposed formation reactions indicates an increase in log*K_e_* from Ln(Tar)(H_4_Spm) to Ln(Tar)(HSpm). This effect suggests the greater stability of complexes containing deprotonated spermine amino groups in the inner coordination sphere of metal ions, along with additional stabilization through non-covalent interactions between the polyamine and α-hydroxy acid.

#### 2.1.4. Ternary Systems of Ln(III)/GA/Spm

Potentiometric studies of the Ln(III)/GA/Spm system were carried out in the range of pH = 2.5–11.0. The values of the overall stability constants (log*β*) of binary systems, as well as protonation constants and metal ion hydrolysis constants, were used to determine the composition of ternary complexes, as well as the values of their overall stability constants (log*β*). The overall stability constants of the complexes formed in the studied systems are shown in Table 3.

The validity of the occurrence of the proposed complex forms of lanthanide ions in the systems studied with glycolic acid and spermine is confirmed by the methods used to verify the data obtained, as described earlier. A comparison of the experimental curve with the computer-generated theoretical curves, as well as the relatively low standard deviations for the overall values of the stability constants of the ternary complexes, confirms the correctness of the choice of complex forms in the Ln(III)/GA/Spm systems.

**Figure 6:**
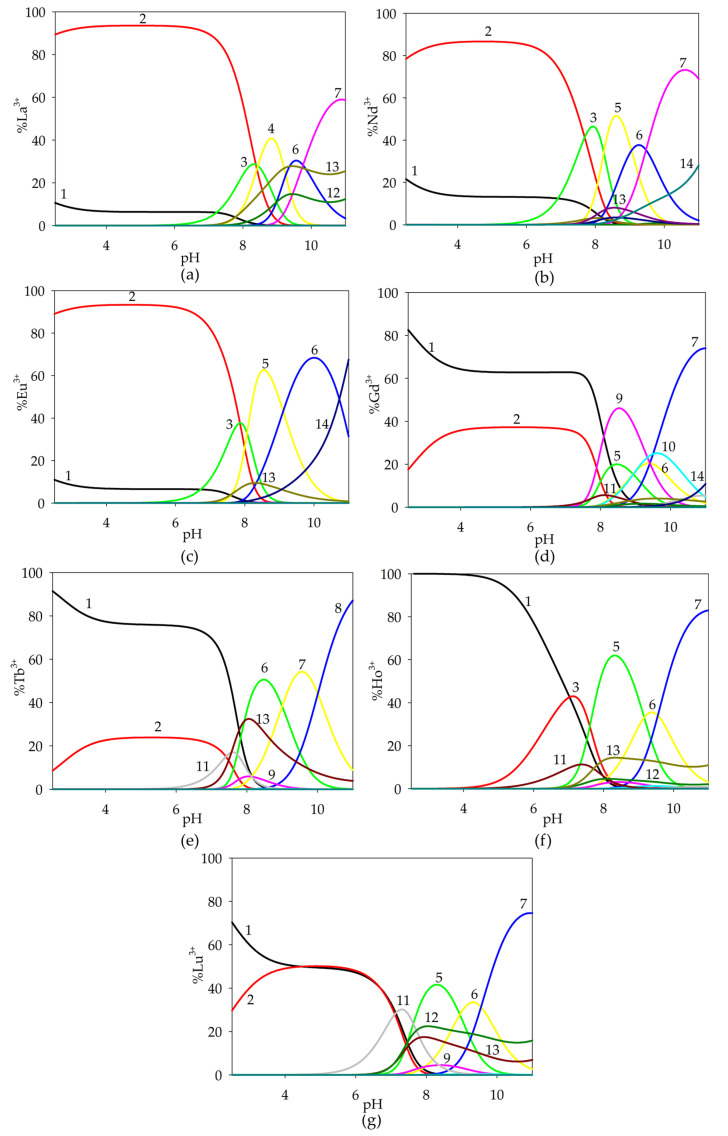
Distribution diagram for the studied systems: (**a**) La(III)/GA/Spm; (**b**) Nd(III)/GA/Spm; (**c**) Eu(III)/GA/Spm; (**d**) Gd(III)/GA/Spm; (**e**) Tb(III)/GA/Spm; (**f**) Ho(III)/GA/Spm; (**g**) Lu(III)/GA/Spm (1—Ln^3+^; 2—Ln(GA)(H_4_Spm); 3—Ln(GA)(H_3_Spm); 4—Ln(GA)(H_2_Spm); 5—Ln(GA)(HSpm); 6—Ln(GA)(Spm); 7—Ln(GA)(Spm)(OH); 8—Ln(GA)(Spm)(OH)_2_; 9—Ln(HSpm); 10—LnSpm; 11—LnGA(OH); 12– LnGA(OH)_2_; 13—Ln(OH)_2_; 14—Ln(OH)_3_). c_metal ion_ = 1 × 10^−3^ mol dm^−3^; c_GA_ = 1 × 10^−3^ mol dm^−3^; c_Spm_= 1 × 10^−3^ mol dm^−3^.

Complexation reactions in ternary systems of spermine and glycolic acid with lanthanide(III) ions begin with the formation of the Ln(GA)(H_4_Spm)-type form (Figure 6). This complex is the dominant form from pH 2.5 to 7.5, and its maximum concentration is 20–90%, depending on the metal ion present in the system. Noteworthy is the fact that the complexation process involves a protonated spermine, which, interacting non-covalently with glycolic acid, occurs in the outer coordination sphere of the central atom. The exception is the system containing holmium(III) ions, where the complexation process begins with the formation of the Ln(GA)(H_3_Spm) form at pH = 4.0.

At pH = 7.0, we observed the formation of several complex compounds that competed with each other. The dominant forms were ternary complexes that bonded up to 60% of the lanthanide(III) ions present in solution. In addition, binary complexes of rare earth ions with glycolic acid and spermine were found at high pH values.

For the Gd(III)/GA/Spm system, there was a marked decrease in log*β* for the observed complex compounds. At high pH values, the dominant forms in this system were binary complexes of the Ln(HSpm) and LnSpm types. Hydroxy complex Gd(GA)(Spm)(OH), which forms from pH = 8.0, reached its maximum concentration at pH = 11.0, binding about 80% of gadolinium(III) ions.

### 2.2. Luminescence Spectroscopy

**Figure 7:**
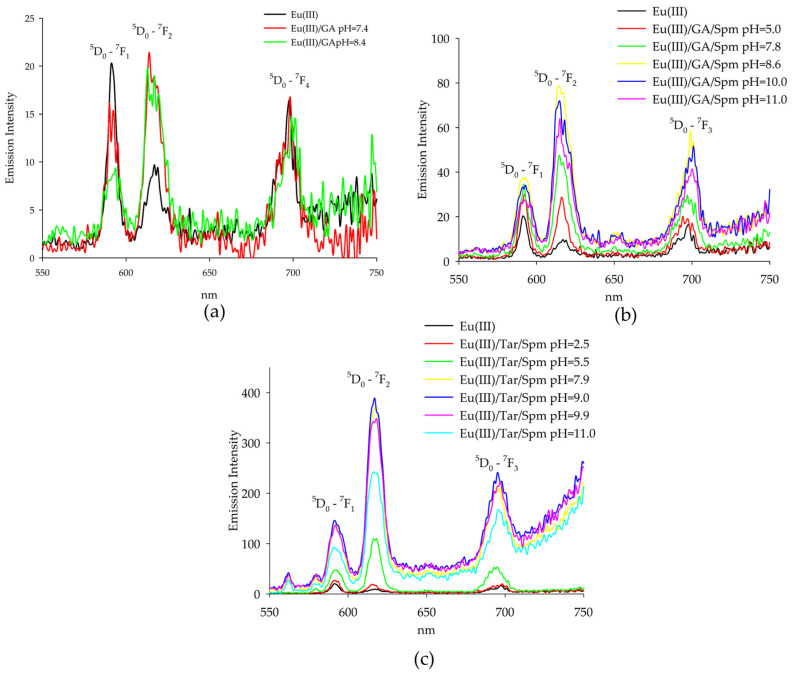
Emission spectra of the following systems: (**a**) Eu(III)/GA; (**b**) Eu(III)/GA/Spm; (**c**) Eu(III)/Tar/Spm.

Luminescence studies were performed at different pH values to determine the dominance of complex compounds in the system, determined from distribution diagrams of the form. Observations of changes in the intensity of characteristic bands in the region of the ^5^D_0_-^7^F_1_, ^7^F_2_ transitions made it possible to determine the effect of glycolic acid on the internal coordination sphere of the lanthanide(III) ion. The emission spectrum of the Eu(III)/GA system was observed at different pH values for the occurrence of complex forms (Figure 7a).

Emission measurements for the glycolic acid system with europium(III) ions showed changes in band intensities for the same wavelengths as the emission maxima of the uncomplexed ions. The increase in band intensity at λ = 618 nm suggests the exchange of water molecules of the aquacomplex Eu(H_2_O)_9_^3+^ by ligand or other molecules. Evidence of these changes is in the increase in the ratio (the ratio of the intensity of the I_em_618/I_em_593 bands), which for the aquacomplex takes a value of 0.47 and increases with the increasing pH of the system, taking values of 0.98 and 1.75. In addition, for the dihydroxyl form, a gradual extinction of luminescence is observed due to the participation of the second hydroxyl group in the coordination of the central atom.

Emission intensity measurements of solutions of Eu(III) complexes with spermine and glycolic acid were carried out, confirming the formation of heteroligand complexes. A strong emission was observed in the region of transitions ^5^D_0_-^7^F_1_, ^7^F_2_, as well as transition ^5^D_0_-^7^F_4_, showing a medium emission intensity when the samples were excited at wavelength λ = 394 nm (Figure 7b). The emission intensity of the ^5^D_0_-^7^F_1_, ^7^F_2_ transitions, similarly to the systems described previously, was affected by the closest surroundings of the ion. It was observed that with increasing pH, the intensity of both bands increased, and at pH = 11.0, there was a decrease in the emission of Eu(III) ions due to the presence of the compound Eu(OH)_3_ in the system. This effect was particularly clearly observed for the band η = 618 nm. The band intensity ratio determined for these solutions (expressed as the ratio η = I_em_618/I_em_593) took the lowest value equal to 0.35 for the aquacomplex, Eu(H_2_O)_9_^3+^. In the presence of ligands and with an increase in pH, the coefficient changed, taking values of 0.78; 1.47; 1.92; 1.86 and 1.74, respectively.

It is noteworthy that at the same pH values, a higher emission intensity of europium(III) ions of the ternary system was observed compared to those of the Eu(III)/Spm [33] and Eu(III)/GA binary systems. This indicates the appearance of the so-called “antenna effect”, in which the polyamine that interacts non-covalently with glycolic acid increases the emission of Eu(III) ions.

The maxima of Eu(III) ion emissions in aqueous solutions containing spermine and tartaric acid occur at the same wavelengths as the maximums of emissions for uncomplexed ions, confirming the influence of ligands on the inner coordination sphere of the central atom. A strong emission is observed in the transition region ^5^D_0_-^7^F_1_, ^7^F_2_ when the samples are excited at a wavelength of λ = 394 nm (Figure 7c).

A significant increase in the intensity of europium(III) emission was observed for the Eu(Tar)(H_4_Spm) complex (pH = 5.5), caused by the presence of completely protonated spermine in the outer coordination sphere of the central atom, interacting non-covalently with the naturally occurring acid. The enhancement of lanthanide emission was induced by the additional interaction of ligands present in the outer coordination sphere. Furthermore, the strongest emission was observed for the complex containing two deprotonated amino groups forming coordination bonds with the metal ion, and two amino groups interacting with the carboxyl groups of tartaric acid. The confirmation of the changes in the closest environment to the central ion was provided by the determined parameter η, which varied with increasing pH as follows: 0.70, 2.31, 2.67, 2.66, 2.73, 2.69.

Emission studies of europium(III) ions showed a significant increase in the emission intensity relative to the binary systems, suggesting the presence of additional interactions affecting the ^5^D_0_-^7^F_1_, ^7^F_2_ transitions and the involvement of additional donor atoms in the complexation process in forming heteroligand forms. In addition, this effect was increasingly observed for the system containing tartaric acid as the α-hydroxy acid.

**Figure 8:**
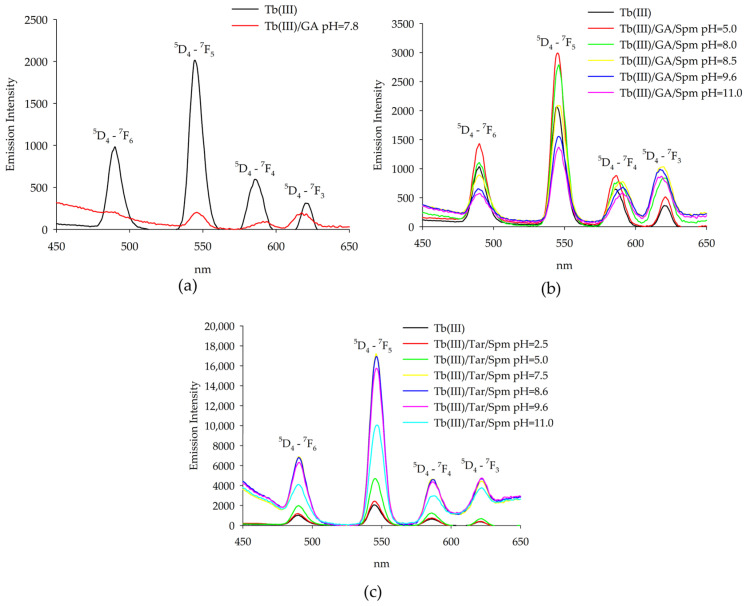
Emission spectra of the following systems: (**a**) Tb(III)/GA; (**b**) Tb(III)/GA/Spm; (**c**) Tb(III)/Tar/Spm.

Changes in the intensity of emission were observed for the terbium(III) system, and emission corresponded to the transitions from the ^5^D_4_ level to the ^7^F_j_ (j = 6, 5, 4, 3) ones, as shown in Figure 8. When the emission intensity for the TbGA(OH) complex was compared with that for free metal ions, a significant reduction was observed, which is due to the presence of a hydroxyl group in the internal coordination sphere. In the ternary Tb(III)/GA/Spm system, a similar relationship was observed for the complexes Tb(GA)(Spm)(OH) and Tb(GA)(Spm)(OH)_2_. The highest increase in intensity was found under conditions where the spermine-protonated amino groups interacted non-covalently with the coordinated acid molecule. In the studied ternary system that contained the terbium(III) ion, tartaric acid and spermine, a significant increase in emission intensity was observed compared to the binary systems and terbium(III) ion alone. The highest emission intensity for the characteristic bands was found for the complex where fully protonated spermine interacted non-covalently with tartaric acid with deprotonated carboxyl groups. In addition, it was found that with the deprotonation of spermine amine groups and their occurrence in the internal coordination sphere, there was a decrease in the intensity of emission.

### 2.3. IR Spectroscopy

**Figure 9:**
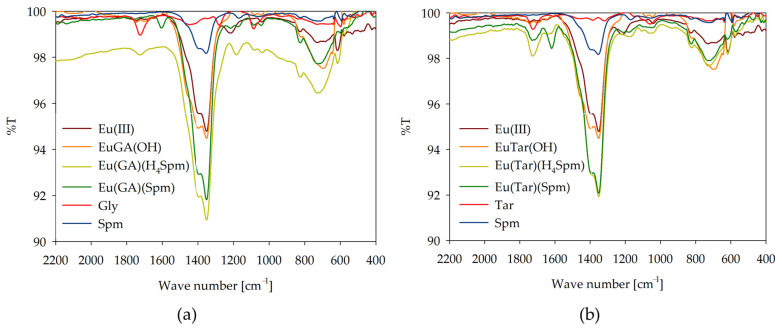
IR spectra of the following systems: (**a**) Eu(III)/GA/Spm; (**b**) Eu(III)/Tar/Spm.

To confirm the mode of coordination, infrared spectroscopy measurements were performed under conditions of dominance of the ternary complex forms on the example of the Eu(III)/GA/Spm and Eu(III)/Tar/Spm systems (Figure 9). The observed changes in the characteristic band corresponding to the stretching vibrations of C=O (~1730 cm^−1^) for the carboxyl groups confirm their involvement in coordination with the lanthanide ions and in the non-covalent interaction with the amino groups of spermine. For binary systems, a quenching of this characteristic band is observed, and for ternary complexes, its amplification is observed. Furthermore, changes are observed in a wave number in the range of 1450–1350 cm^−1^, associated with symmetric stretching vibrations of the carboxylate group in α-hydroxy acid, modified by interactions with europium(III) ions. Notable is the fact that for the Ln(GA)(Spm) and Ln(Tar)(Spm) complexes, an additional band is observed at a wave number of ~1605 cm^−1^. This band corresponds to the deformation vibrations of the amine groups of spermine, which under these conditions are fully deprotonated and occur in the inner coordination sphere of the metal ion. The lack of observations of this band at pH values corresponding to the complexes containing polyamine with protonated amine groups may suggest their occurrence in the external coordination sphere and their interaction with the negatively charged carboxyl groups of the acid molecule.

## 3. Materials and Methods

### 3.1. Materials

Glycolic acid (GA) and L-Tartaric acid (Tar) were obtained from Merck (Darmstadt, Germany) and used without further purification. Spermine (Spm) was obtained by mixing one chemical equivalent of the amine suspended in methanol with the chemical equivalents required from a HNO_3_ solution of water and methanol. The white salts were recrystallized, washed with methanol and dried in a desiccator on P_4_O_10_. The synthesis of spermine tetranitrate (Spm·4HNO₃) was performed to neutralize the strong basicity of free spermine, which poses significant challenges in establishing and maintaining a defined initial pH. In its free form, spermine is a highly basic compound that readily binds protons, potentially leading to uncontrolled pH shifts, especially in the acidic range. Its nitrate salt form not only reduces this effect but also facilitates thermodynamic measurements under low pH conditions (starting at pH 2.5), ensuring the greater stability and reproducibility of the experimental setup. Lanthanide(III) nitrates (La(III), Nd(III), Gd(III), Tb(III), Ho(III) and Lu(III)) were obtained from Sigma-Aldrich (Darmstadt, Germany) and used without further purification.

### 3.2. Equilibrium Study

The thermodynamic study equipment used, the way it was prepared for testing, the measurement conditions used, and the way calculations were carried out are described in detail in our earlier work [32,33]. A Titrando 905 Metrohm equipped with an autoburette with a Metrohm 6.0233.100 electrode (Metrohm AG, Herisau, Switzerland) was used for potentiometric titrations. Before each titration, the electrode was calibrated in terms of the concentration of hydrogen ions [34,35]. The pH meter indication was corrected by two standard buffer solutions of pH 4.002 and pH 9.225 before each series of measurements. Each titration was carried out under strict measurement conditions: a constant ionic strength of 0.1M (KNO_3_), a constant temperature of 20 ± 1 °C (titration dish placed in thermostatic bath set at this temperature), an atmosphere of neutral gas (helium—Ultra High Purity 5.0), titrant CO_2_-free NaOH with a well-defined concentration (titrant addition step—0.006 mL) and a pH ranging from 2.5 to 11.0. The measurements were taken in metal-to-ligand molar ratios of 1:1 in the binary systems and 1:1:1 in the ternary systems, where the concentration of metal ions was 0.001M; the ligand-to-ligand molar ratio was 1:1 in the binary system (without metal ion) and the concentrations of ligands were 0.001M. The data corresponding to the curves selected to generate the distribution diagrams and the corresponding titration curve plots are presented in the Supplementary Materials (Tables S1–S5; Figures S1–S5). The protonation constants and stability constants of the complexes were determined with HYPERQUAD2008 using the data obtained from each titration [36,37]. The iteration procedure allowed the types (stoichiometry) and thermodynamic stability of the complexes formed in the studied systems to be determined. The correctness of the assumed model was verified by analyzing the standard deviation, comparing the convergence of the experimental curve to that obtained from the model, and evaluating it using the Hamilton test and the chi-square test. The computer program used the non-linear method of least squares to minimize the sum (*S*) of the squares of residuals between the observed quantities (*f^obs^*) and those calculated on the basis of the model (*f^calc^*).

$$S=\sum_{i=1}^{n}w_{i}{\left(f_{i}^{obs}-f_{i}^{calc}\right)}^{2}$$

*n*—number of measurements; *w_i_*—statistical weight.

The following equilibriums were evaluated as stability constants of the studied systems: binary systems with metal ion (1) oM + pL + qH ⇆ M_o_L_p_H_q_ (where M = metal ion; L = ligand); binary systems without metal ion (2) oL + pSpm + qH ⇆ L_o_Spm_p_H_q_; ternary systems (3) oM + pL + qSpm + rH ⇆ M_o_L_p_Spm_q_H_r_. They were then calculated using the following equations:

(1) $$\beta =\frac{M_{o}L_{p}H_{q}}{{\left[M\right]}^{o}{\left[L\right]}^{p}{\left[H\right]}^{q}}$$

(2) $$\beta =\frac{L_{o}{Spm}_{p}H_{q}}{{\left[L\right]}^{o}{\left[Spm\right]}^{p}{\left[H\right]}^{q}}$$

(3) $$\beta =\frac{M_{o}L_{p}{Spm}_{q}H_{r}}{{\left[M\right]}^{o}{\left[L\right]}^{p}{\left[Spm\right]}^{q}{\left[H\right]}^{r}}$$

For hydroxo complexes, the *β* values were defined as follows:

(4) $$\beta =\frac{M_{o}L_{p}{\left(OH\right)}_{q}}{{\left[M\right]}^{o}{\left[L\right]}^{p}{\left[OH\right]}^{q}}$$

(5) $$\beta =\frac{M_{o}L_{p}{Spm}_{q}{\left(OH\right)}_{r}}{{\left[M\right]}^{o}{\left[L\right]}^{p}{\left[Spm\right]}^{q}{\left[OH\right]}^{r}}$$

where M, Spm, H^+^ and OH^−^ represent the metal ion, ligand, proton and hydroxide ion, respectively. In this definition, the hydroxido complexes are described in terms of hydroxide ion participation, and not solely proton balance.

To provide a more intuitive comparison of protonation or deprotonation steps between species, apparent equilibrium constants (log*K_e_*) were calculated and presented where appropriate. These constants were derived from log*β* values using the following relationship:

log*K* = log*β* − *n*p*K_w_*

Here, *n* denotes the number of hydroxide ions involved in the reaction step, and p*K_w_* = 13.76 corresponds to the ionic product of water (*K_w_*) at the experimental temperature and ionic strength.

In our calculations, the activity of water is considered constant and equal to 1, as is standard in aqueous equilibria studies. Therefore, it is not explicitly included in the equilibrium expressions. The value of pK_w_ used does not represent the dissociation constant (pK_a_) of water but refers to the following:

H_2_O ⇌ H^+^ + OH^−^, K_w_ = [H^+^][OH^−^]

For binary and ternary hydroxo species, the equilibrium constants correspond to the following reactions:

M + L ⇆ ML(OH) + H^+^; M + L + Spm ⇆ MLSpm(OH)

where

*β_ML_* = [ML(OH)]/[M][L][OH^−^]; *β*_MLSpm_ = [MLSpm(OH)]/[M][L][Spm][OH^−^]

### 3.3. Luminescence Spectroscopy

The samples were prepared in ultra-high-quality water using a Simplicity Ultrapure Water System (Millipore, Darmstadt, Germany). The concentration of metal ions was 0.001M. The luminescence studies were recorded on an RF-600 spectrofluorophotometer (Schimadzu, Kyoto, Japan). Terbium(III) ion samples were excited at 370 nm using 10.0/5.0 nm slit widths, and solutions of europium(III) ion systems were excited at 395 nm with 3.0/3.0 nm slit widths.

### 3.4. Infrared Spectroscopy (FT-IR)

Infrared spectra were recorded on an FT-IR INVENIO R spectrophotometer (Bruker, Bremen, Germany). Samples were prepared by dissolving the compounds studied in D_2_O. The metal and ligand concentration for the IR studies was 0.1 M. The pH values were adjusted by the addition of NaOD or DCl. pH values were corrected according to the formula pD = pH reading + 0.4 [34].

## 4. Conclusions

In binary systems using the potentiometric method, only hydroxy complexes were found in the system containing lanthanide ions and glycolic acid, while in the system of glycolic acid with spermine, non-covalent interactions were found between the tested ligands and the formation of three adducts. Ternary systems showed a difference in the formation of complex forms depending on the studied α-hydroxy acid. Studies of binary systems of spermine with prussic acid showed an increase in the contribution of bioligands to the formation of adducts for tartaric acid, which contained additional potential negative interaction centers in the structure. Tartaric acid with the studied lanthanide ions and spermine only formed compounds of the type Ln(H_x_Tar)(H_y_Spm) (where x = 1 or 0; y = 1–4), whereas for the ternary system containing glycolic acid, the formation of LnGAH_x_Spm-type complexes (where x = 0–4) and hydroxy complexes Ln(GA)(Spm)(OH)_x_ (where x = 1–2) was observed. For ternary systems, the formation of coordination bonds of lanthanide ions with oxygen atoms of deprotonated carboxyl groups of α-hydroxy acids was found at low pH values. The appearance of protonated polyamine was observed in the outer coordination sphere of the central atom that interacts with α-hydroxy acid molecules. With an increase in the pH, a gradual rupture of non-covalent interactions and direct participation in the coordination of deprotonated nitrogen atoms of amine groups was observed in the systems studied. Changes in the coordination sphere were confirmed by using spectroscopic methods. Luminescence studies confirmed the presence of amino groups in the outer coordination sphere in ternary systems where amino groups interact non-covalently with the carboxyl groups of α-hydroxy acids. The so-called “antenna effect” observed was greater for systems containing tartaric acid. In addition, at higher pH values, luminescence extinction was observed during the formation of hydroxy complexes, which confirmed the presence of a hydroxyl group in the internal coordination sphere. In addition, infrared spectroscopy studies have shown that at an alkaline pH, deprotonated amine groups occur in the inner coordination sphere of the central atom, as evidenced by the appearance of deformation vibrations of the amine groups (~1605 cm^−1^).

## Figures and Tables

**Table 1:** Overall stability constants (log*β*) and equilibrium constants of formation (log*K_e_*) of the binary complexes in the system of Ln(III)/GA (standard deviations are given in parentheses).

Species	Overall Stability Constants (log*β*)	Reaction	Equilibrium Constants (log*K_e_*)
LaGA(OH)_2_	−12.36 (1)	La^3+^ + GA^−^ + 2H_2_O ⇆ LaGA(OH)_2_ + 2H^+^	15.17
NdGA(OH)	−4.28 (6)	Nd^3+^ + GA^−^ + H_2_O ⇆ [NdGA(OH)]^+^ + H^+^	9.48
NdGA(OH)_2_	−12.54 (6)	[NdGA(OH)]^+^ + H_2_O ⇆ NdGA(OH)_2_ + H^+^	5.50
EuGA(OH)	−4.25 (9)	Eu^3+^ + GA^−^ + H_2_O ⇆ [EuGA(OH)]^+^ + H^+^	9.51
EuGA(OH)_2_	−11.81 (4)	[EuGA(OH)]^+^ + H_2_O ⇆ EuGA(OH)_2_ + H^+^	6.21
GdGA(OH)	−5.70 (6)	Gd^3+^ + GA^−^ + H_2_O ⇆ [GdGA(OH)]^+^ + H^+^	8.06
GdGA(OH)_2_	−14.84 (3)	Gd^3+^ + GA^−^ + 2H_2_O ⇆ GdGA(OH)_2_ + 2H^+^	4.62
TbGA(OH)	−4.81 (7)	Tb^3+^ + GA^−^ + 2H_2_O ⇆ TbGA(OH)_2_ + 2H^+^	8.95
HoGA(OH)	−4.31 (7)	Ho^3+^ + GA^−^ + H_2_O ⇆ [HoGA(OH)]^+^ + H^+^	9.45
HoGA(OH)_2_	−12.41 (7)	[HoGA(OH)]^+^ + H_2_O ⇆ HoGA(OH)_2_ + H^+^	5.67
LuGA(OH)	−3.68 (7)	Lu^3+^ + GA^−^ + H_2_O ⇆ [LuGA(OH)]^+^ + H^+^	10.08
LuGA(OH)_2_	−11.49 (7)	[LuGA(OH)]^+^ + H_2_O ⇆ LuGA(OH)_2_ + H^+^	5.95

Hydrolysis constants used in calculations: La(OH)_2_ = −15.54; Nd(OH)_2_ = −16.12; Nd(OH)_3_ = −25.53; Eu(OH)_2_ = −15.15; Eu(OH)_3_ = −24.31; Gd(OH) = −9.30; Gd(OH)_2_ = −17.74; Gd(OH)_3_ = −28.12; Tb(OH)_2_ = −15.56; Ho(OH)_2_ = −15.62; Lu(OH)_2_ = −14.93.

**Table 2:** Composition, stability constants (log*β*) and overall equilibrium constants of complex formation reactions (log*K_e_*) in the Ln(III)/Tar/Spm system.

Species	Overall Stability Constants (log*β*)	Reaction	Equilibrium Constants (log*K_e_*)
La(HTar)(H_4_Spm)	54.26 (5)	[LaHTar]^2+^ + H_4_Spm ⇆ [La(HTar)]^2+^·(H_4_Spm)	7.49
La(Tar)(H_4_Spm)	49.03 (4)	[LaTar]^+^ + H_4_Spm ⇆ [La(Tar)]^+^·(H_4_Spm)	6.01
La(Tar)(H_3_Spm)	41.44 (3)	[LaTar]^+^ + H_3_Spm^−^ ⇆ [La(Tar)(H_3_Spm)]	6.70
La(Tar)(H_2_Spm)	32.86 (3)	[LaTar]^+^ + H_2_Spm^2−^ ⇆ [La(Tar)(H_2_Spm)]^−^	7.23
La(Tar)(HSpm)	23.69 (3)	[LaTar]^+^ + HSpm^3−^ ⇆ [La(Tar)(HSpm)]^2−^	8.43
Nd(HTar)(H_4_Spm)	52.80 (5)	Nd^3+^ + HTar^−^ + H_4_Spm ⇆ [Nd(HTar)]^2+^·(H_4_Spm)	9.22
Nd(Tar)(H_4_Spm)	48.67 (5)	Nd^3+^ + [(Tar)(H_4_Spm)]^2−^ ⇆ [Nd(Tar)]^+^·(H_4_Spm)	6.52
Nd(Tar)(H_3_Spm)	41.78 (3)	Nd^3+^ + [(Tar)(H_3_Spm)]^3−^ ⇆ [Nd(Tar)(H_3_Spm)]	8.33
Nd(Tar)(H_2_Spm)	32.97 (4)	Nd^3+^ + [(Tar)(H_2_Spm)]^4−^ ⇆ [Nd(Tar)(H_2_Spm)]^−^	8.53
Nd(Tar)(HSpm)	24.32 (2)	Nd^3+^ + [(Tar)(HSpm)]^5−^ ⇆ [Nd(Tar)(HSpm)]^2−^	9.88
Eu(HTar)(H_4_Spm)	54.52 (5)	Eu^3+^ + HTar^−^ + H_4_Spm ⇆ [Eu(HTar)]^2+^·(H_4_Spm)	10.94
Eu(Tar)(H_4_Spm)	50.44 (4)	Eu^3+^ + [(Tar)(H_4_Spm)]^2−^ ⇆ [Eu(Tar)]^+^·(H_4_Spm)	8.29
Eu(Tar)(H_3_Spm)	43.63 (3)	Eu^3+^ + [(Tar)(H_3_Spm)]^3−^ ⇆ [Eu(Tar)(H_3_Spm)]	10.18
Eu(Tar)(H_2_Spm)	34.70 (4)	Eu^3+^ + [(Tar)(H_2_Spm)]^4−^ ⇆ [Eu(Tar)(H_2_Spm)]^−^	10.26
Eu(Tar)(HSpm)	25.97 (3)	Eu^3+^ + [(Tar)(HSpm)]^5−^ ⇆ [Eu(Tar)(HSpm)]^2−^	11.53
Tb(HTar)(H_4_Spm)	51.13 (8)	Tb^3+^ + HTar^−^ + H_4_Spm ⇆ [Tb(HTar)]^2+^·(H_4_Spm)	7.55
Tb(Tar)(H_4_Spm)	47.58 (6)	Tb^3+^ + [(Tar)(H_4_Spm)]^2−^ ⇆ [Tb(Tar)]^+^·(H_4_Spm)	5.43
Tb(Tar)(H_3_Spm)	41.13 (4)	Tb^3+^ + [(Tar)(H_3_Spm)]^3−^ ⇆ [Tb(Tar)(H_3_Spm)]	7.68
Tb(Tar)(H_2_Spm)	32.48 (6)	Tb^3+^ + [(Tar)(H_2_Spm)]^4−^ ⇆ [Tb(Tar)(H_2_Spm)]^−^	8.04
Tb(Tar)(HSpm)	23.84 (4)	Tb^3+^ + [(Tar)(HSpm)]^5−^ ⇆ [Tb(Tar)(HSpm)]^2−^	9.40
Ho(HTar)(H_4_Spm)	50.78 (3)	Ho^3+^ + HTar^−^ + H_4_Spm ⇆ [Ho(HTar)]^2+^·(H_4_Spm)	7.20
Ho(Tar)(H_4_Spm)	46.99 (3)	Ho^3+^ + [(Tar)(H_4_Spm)]^2−^ ⇆ [Ho(Tar)]^+^·(H_4_Spm)	4.84
Ho(Tar)(H_3_Spm)	40.75 (1)	Ho^3+^ + [(Tar)(H_3_Spm)]^3−^ ⇆ [Ho(Tar)(H_3_Spm)]	7.3
Ho(Tar)(H_2_Spm)	31.97 (4)	Ho^3+^ + [(Tar)(H_2_Spm)]^4−^ ⇆ [Ho(Tar)(H_2_Spm)]^−^	7.53
Lu(HTar)(H_4_Spm)	52.92 (2)	Lu^3+^ + HTar^−^ + H_4_Spm ⇆ [Lu(HTar)]^2+^·(H_4_Spm)	9.24
Lu(Tar)(H_4_Spm)	49.06 (1)	Lu^3+^ + [(Tar)(H_4_Spm)]^2−^ ⇆ [Lu(Tar)]^+^·(H_4_Spm)	6.91
Lu(Tar)(H_3_Spm)	42.18 (1)	Lu^3+^ + [(Tar)(H_3_Spm)]^3−^ ⇆ [Lu(Tar)(H_3_Spm)]	8.73
Lu(Tar)(H_2_Spm)	33.38 (1)	Lu^3+^ + [(Tar)(H_2_Spm)]^4−^ ⇆ [Lu(Tar)(H_2_Spm)]^−^	8.94
Lu(Tar)(HSpm)	24.01 (2)	Lu^3+^ + [(Tar)(HSpm)]^5−^ ⇆ [Lu(Tar)(HSpm)]^2−^	9.57

Hydrolysis constants used in calculations: La(OH)_2_ = −15.54; Nd(OH)_2_ = −16.12; Nd(OH)_3_ = −25.53; Eu(OH)_2_ = −15.15; Eu(OH)_3_ = −24.31; Gd(OH) = −9.30; Gd(OH)_2_ = −17.74; Gd(OH)_3_ = −28.12; Tb(OH)_2_ = −15.56; Ho(OH)_2_ = −15.62; Lu(OH)_2_ = −14.93.

**Table 3:** Composition, stability constants (log*β*) and overall equilibrium constants of complex formation reactions (log*K_e_*) in the following system: Ln(III)/GA/Spm.

Species	Overall Stability Constants (log*β*)	Reaction	Equilibrium Constants (log*K_e_*)
La(GA)(H_4_Spm)	48.23 (2)	La^3+^ + GA^−^ + H_4_Spm ⇆ [La(GA)]^2+^·(H_4_Spm)	9.56
La(GA)(H_3_Spm)	39.85 (2)	La^3+^ + [(GA)(H_3_Spm)]^2−^ ⇆ [La(GA)(H_3_Spm)]^+^	6.76
La(GA)(H_2_Spm)	31.41 (2)	La^3+^ + [(GA)(H_2_Spm)]^3−^ ⇆ [La(GA)(H_2_Spm)]	6.79
La(GA)(Spm)	12.90 (5)	[LaSpm]^−^ + GA^−^ ⇆ [La(GA)(Spm)]^2−^	6.75
La(GA)(Spm)(OH)	3.14 (3)	[La(GA)(Spm)]^2−^ + H_2_O ⇆ [La(GA)(Spm)(OH)]^3−^ + H^+^	4.01
Nd(GA)(H_4_Spm)	47.25 (8)	Nd^3+^ + GA^−^ + H_4_Spm ⇆ [Nd(GA)]^2+^·(H_4_Spm)	8.58
Nd(GA)(H_3_Spm)	39.56 (9)	Nd^3+^ + [(GA)(H_3_Spm)]^2−^ ⇆ [Nd(GA)(H_3_Spm)]^+^	6.47
Nd(GA)(HSpm)	23.07 (9)	Nd^3+^ + [(GA)(HSpm)]^4−^ ⇆ [Nd(GA)(HSpm)]^−^	7.96
Nd(GA)(Spm)	14.02 (8)	[NdSpm]^−^ + GA^−^ ⇆ [Nd(GA)(Spm)]^2−^	6.31
Nd(GA)(Spm)(OH)	4.55 (9)	[Nd(GA)(Spm)]^2−^ + H_2_O ⇆ [Nd(GA)(Spm)(OH)]^3−^ + H^+^	4.30
Eu(GA)(H_4_Spm)	48.19 (3)	Eu^3+^ + GA^−^ + H_4_Spm ⇆ [Eu(GA)]^2+^·(H_4_Spm)	9.52
Eu(GA)(H_3_Spm)	40.33 (4)	Eu^3+^ + [(GA)(H_3_Spm)]^2−^ ⇆ [Eu(GA)(H_3_Spm)]^+^	7.24
Eu(GA)(HSpm)	24.20 (3)	Eu^3+^ + [(GA)(HSpm)]^4−^ ⇆ [Eu(GA)(HSpm)]^−^	9.09
Eu(GA)(Spm)	15.09 (3)	[EuSpm]^−^ + GA^−^ ⇆ [Eu(GA)(Spm)]^2−^	6.74
Gd(GA)(H_4_Spm)	44.85 (5)	Gd^3+^ + GA^−^ + H_4_Spm ⇆ [Gd(GA)]^2+^·(H_4_Spm)	6.18
Gd(GA)(HSpm)	20.84 (7)	Gd^3+^ + [(GA)(HSpm)]^4−^ ⇆ [Gd(GA)(HSpm)]^−^	5.73
Gd(GA)(Spm)	11.91 (7)	[GdSpm]^−^ + GA^−^ ⇆ [Gd(GA)(Spm)]^2−^	3.23
Gd(GA)(Spm)(OH)	2.50 (4)	[Gd(GA)(Spm)]^2−^ + H_2_O ⇆ [Gd(GA)(Spm)(OH)]^3−^ + H^+^	4.35
Tb(GA)(H_4_Spm)	44.41 (6)	Tb^3+^ + GA^−^ + H_4_Spm ⇆ [Tb(GA)]^2+^·(H_4_Spm)	5.74
Tb(GA)(Spm)	13.93 (2)	[TbSpm]^−^ + GA^−^ ⇆ [Tb(GA)(Spm)]^2−^	5.96
Tb(GA)(Spm)(OH)	4.93 (1)	[Tb(GA)(Spm)]^2−^ + H_2_O ⇆ [Tb(GA)(Spm)(OH)]^3−^ + H^+^	4.77
Tb(GA)(Spm)(OH)_2_	−5.08 (2)	[Tb(GA)(Spm)(OH)]^3−^ + H_2_O ⇆ [Tb(GA)(Spm)(OH)_2_]^4−^ + H^+^	3.74
Ho(GA)(H_3_Spm)	38.29 (4)	Ho^3+^ + [(GA)(H_3_Spm)]^2−^ ⇆ [Ho(GA)(H_3_Spm)]^+^	5.20
Ho(GA)(HSpm)	23.08 (2)	Ho^3+^ + [(GA)(HSpm)]^4−^ ⇆ [Ho(GA)(HSpm)]^−^	7.38
Ho(GA)(Spm)	13.92 (3)	[HoSpm]^−^ + GA^−^ ⇆ [Ho(GA)(Spm)]^2−^	5.27
Ho(GA)(Spm)(OH)	4.37 (3)	[Ho(GA)(Spm)]^2−^ + H_2_O ⇆ [Ho(GA)(Spm)(OH)]^3−^ + H^+^	4.21
Lu(GA)(H_4_Spm)	45.29 (4)	Lu^3+^ + [(GA)(H_4_Spm)]^2−^ ⇆ [Lu(GA)]^2+^·(H_4_Spm)	6.62
Lu(GA)(HSpm)	22.99 (3)	Lu^3+^ + [(GA)(HSpm)]^4−^ ⇆ [Lu(GA)(HSpm)]^−^	7.88
Lu(GA)(Spm)	14.04 (3)	[LuSpm]^−^ + GA^−^ ⇆ [Lu(GA)(Spm)]^2−^	5.55
Lu(GA)(Spm)(OH)	4.49 (3)	[Lu(GA)(Spm)]^2−^ + H_2_O ⇆ [Lu(GA)(Spm)(OH)]^3−^ + H^+^	4.21

Hydrolysis constants used in calculations: La(OH)_2_ = −15.54; Nd(OH)_2_ = −16.12; Nd(OH)_3_ = −25.53; Eu(OH)_2_ = −15.15; Eu(OH)_3_ = −24.31; Gd(OH) = −9.30; Gd(OH)_2_ = −17.74; Gd(OH)_3_ = −28.12; Tb(OH)_2_ = −15.56; Ho(OH)_2_ = −15.62; Lu(OH)_2_ = −14.93.

## Data Availability

All data generated or analyzed during this study are included in this published article (and its Supplementary Materials).

## Supplementary Materials

The supporting information can be downloaded at: https://www.mdpi.com/article/10.3390/ijms26104477/s1.
