# Supplementary material for: Reactions of Lanthanide Ions with Glycolic Acid or Tartaric Acid in the Presence of Spermine: Potentiometric and Spectroscopic Studies
Source: Int J Mol Sci. 2025 May 8;26(10):4477. doi: 10.3390/ijms26104477 (PMC12111230; doi:10.3390/ijms26104477)
Supplement: Supplementary file 1 [file ijms-26-04477-s001.zip › ijms-3523835-supplementary.pdf]

Table S1. Data obtained from potentiometric measurements during glycolic acid protonation studies.

V [ml]	pH
	GA
0.0000	2.4446
0.0080	2.4396
0.0160	2.4536
0.0240	2.4566
0.0320	2.4686
0.0400	2.4776
0.0480	2.4856
0.0560	2.4936
0.0640	2.4996
0.0720	2.5116
0.0800	2.5206
0.0880	2.5296
0.0960	2.5376
0.1040	2.5466
0.1120	2.5566
0.1200	2.5656
0.1280	2.5756
0.1360	2.5846
0.1440	2.5946
0.1520	2.6036
0.1600	2.6136
0.1680	2.6246
0.1760	2.6346
0.1840	2.6446
0.1920	2.6536
0.2000	2.6676
0.2080	2.6776
0.2160	2.6896
0.2240	2.7016
0.2320	2.7016
0.2400	2.7256
0.2480	2.7386
0.2560	2.7516
0.2640	2.7646
0.2720	2.7796
0.2800	2.7946
0.2880	2.8086
0.2960	2.8226
0.3040	2.8366
0.3120	2.8536
0.3200	2.8696
0.3280	2.8856
0.3360	2.9026
0.3440	2.9186
0.3520	2.9376
0.3600	2.9556
0.3680	2.9736

0.3760	2.9926
0.3840	3.0146
0.3920	3.0336
0.4000	3.0576
0.4080	3.0756
0.4160	3.0966
0.4240	3.1226
0.4320	3.1496
0.4400	3.1756
0.4480	3.2026
0.4560	3.2296
0.4640	3.2586
0.4720	3.2896
0.4800	3.3156
0.4880	3.3516
0.4960	3.3836
0.5040	3.4196
0.5120	3.4536
0.5200	3.4896
0.5280	3.5276
0.5360	3.5656
0.5440	3.6056
0.5520	3.6496
0.5600	3.6936
0.5680	3.7406
0.5760	3.7876
0.5840	3.8386
0.5920	3.8906
0.6000	3.9476
0.6080	4.0086
0.6160	4.0726
0.6240	4.1436
0.6320	4.2226
0.6400	4.3126
0.6480	4.4196
0.6560	4.5506
0.6640	4.7256
0.6720	4.9856
0.6800	5.5916
0.6880	7.9666
0.6960	9.1266
0.7040	9.5096
0.7120	9.7726
0.7200	9.9676
0.7280	10.1136
0.7360	10.2336
0.7440	10.3336
0.7520	10.4116
0.7600	10.4846
0.7680	10.5476
0.7760	10.6016

0.7840	10.6496
0.7920	10.6916
0.8000	10.7336
0.8080	10.7676
0.8160	10.8026
0.8240	10.8336
0.8320	10.8606
0.8400	10.8896
0.8480	10.9166
0.8560	10.9386

Table S2. Data obtained from potentiometric measurements during studies of lanthanide ion(III)/glycolic acid binary systems.

V [ml]	pH						
	**La(III)**	Nd(III)/GA	**La(III)**	Gd(III)/GA	**La(III)**	Ho(III)/GA	**La(III)**
0.0000	2.4641	2.4658	2.4438	2.5098	2.3008	2.3096	2.4126
0.0060	2.4691	2.4748	2.4488	2.4968	2.2988	2.3186	2.4046
0.0120	2.4761	2.4808	2.4558	2.5018	2.2998	2.3276	2.4086
0.0180	2.4831	2.4898	2.4648	2.5068	2.3028	2.3326	2.4116
0.0240	2.4901	2.4988	2.4748	2.5128	2.3058	2.3396	2.4156
0.0300	2.4991	2.5078	2.4828	2.5198	2.3088	2.3446	2.4186
0.0360	2.5051	2.5158	2.4918	2.5288	2.3118	2.3506	2.4226
0.0420	2.5141	2.5248	2.5008	2.5358	2.3148	2.3566	2.4256
0.0480	2.5211	2.5338	2.5108	2.5428	2.3188	2.3616	2.4296
0.0540	2.5321	2.5418	2.5218	2.5508	2.3218	2.3686	2.4336
0.0600	2.5401	2.5528	2.5308	2.5578	2.3258	2.3736	2.4366
0.0660	2.5491	2.5608	2.5408	2.5668	2.3178	2.3786	2.4406
0.0720	2.5571	2.5698	2.5498	2.5738	2.3338	2.3846	2.4446
0.0780	2.5661	2.5788	2.5578	2.5818	2.3378	2.3896	2.4486
0.0840	2.5761	2.5888	2.5678	2.5898	2.3418	2.3926	2.4516
0.0900	2.5851	2.6028	2.5768	2.5988	2.3458	2.3976	2.4546
0.0960	2.5941	2.6098	2.5858	2.6068	2.3508	2.4026	2.4586
0.1020	2.6041	2.6198	2.5948	2.6158	2.3418	2.4086	2.4616
0.1080	2.6141	2.6288	2.6048	2.6238	2.3468	2.4116	2.4676
0.1140	2.6251	2.6378	2.6148	2.6328	2.3528	2.4166	2.4696
0.1200	2.6351	2.6478	2.6248	2.6418	2.3568	2.4226	2.4736
0.1260	2.6451	2.6578	2.6348	2.6508	2.3608	2.4256	2.4786
0.1320	2.6561	2.6688	2.6448	2.6608	2.3658	2.4306	2.4836
0.1380	2.6661	2.6788	2.6548	2.6698	2.3718	2.4356	2.4866
0.1440	2.6781	2.6888	2.6658	2.6798	2.3748	2.4396	2.4916
0.1500	2.6891	2.6978	2.6768	2.6898	2.3798	2.4446	2.4946
0.1560	2.7011	2.7098	2.6868	2.6998	2.3848	2.4476	2.4986
0.1620	2.7131	2.7208	2.6968	2.7108	2.3888	2.4536	2.5036
0.1680	2.7251	2.7318	2.7098	2.7208	2.3938	2.4566	2.5076
0.1740	2.7391	2.7428	2.7208	2.7318	2.3988	2.4616	2.5116
0.1800	2.7531	2.7548	2.7318	2.7428	2.4088	2.4566	2.5166
0.1860	2.7671	2.7658	2.7438	2.7548	2.4148	2.4706	2.5206
0.1920	2.7801	2.7778	2.7558	2.7648	2.4198	2.4756	2.5256

0.1980	2.7941	2.7908	2.7688	2.7768	2.4248	2.4796	2.5286
0.2040	2.8081	2.8038	2.7818	2.7878	2.4288	2.4846	2.5336
0.2100	2.8221	2.8158	2.7948	2.7998	2.4338	2.4786	2.5376
0.2160	2.8371	2.8288	2.8078	2.8118	2.4388	2.4916	2.5426
0.2220	2.8531	2.8418	2.8208	2.8248	2.4438	2.4966	2.5466
0.2280	2.8681	2.8558	2.8368	2.8378	2.4408	2.4926	2.5496
0.2340	2.8851	2.8718	2.8518	2.8508	2.4458	2.4986	2.5566
0.2400	2.9011	2.8838	2.8658	2.8638	2.4508	2.5036	2.5616
0.2460	2.9191	2.8998	2.8808	2.8778	2.4558	2.5076	2.5656
0.2520	2.9361	2.9128	2.8948	2.8928	2.4608	2.5116	2.5706
0.2580	2.9551	2.9268	2.9108	2.9078	2.4668	2.5176	2.5756
0.2640	2.9741	2.9408	2.9258	2.9228	2.4718	2.5226	2.5796
0.2700	2.9931	2.9548	2.9418	2.9378	2.4768	2.5276	2.5846
0.2760	3.0131	2.9718	2.9578	2.9538	2.4818	2.5326	2.5916
0.2820	3.0341	2.9878	2.9758	2.9698	2.4868	2.5376	2.5946
0.2880	3.0551	3.0038	2.9938	2.9908	2.4938	2.5426	2.5996
0.2940	3.0781	3.0218	3.0118	3.0078	2.4988	2.5486	2.6056
0.3000	3.1001	3.0378	3.0308	3.0258	2.5048	2.5536	2.6106
0.3060	3.1241	3.0568	3.0498	3.0428	2.5168	2.5586	2.6156
0.3120	3.1491	3.0748	3.0708	3.0608	2.5208	2.5636	2.6206
0.3180	3.1741	3.0948	3.0898	3.0798	2.5208	2.5686	2.6246
0.3240	3.2011	3.1148	3.1108	3.0988	2.5268	2.5746	2.6316
0.3300	3.2291	3.1348	3.1328	3.1188	2.5318	2.5856	2.6366
0.3360	3.2581	3.1548	3.1548	3.1388	2.5388	2.5916	2.6416
0.3420	3.2881	3.1768	3.1778	3.1598	2.5498	2.5956	2.6466
0.3480	3.3201	3.1978	3.2008	3.1808	2.5558	2.6016	2.6516
0.3540	3.3531	3.2218	3.2258	3.2028	2.5618	2.6076	2.6576
0.3600	3.3871	3.2458	3.2518	3.2258	2.5678	2.6056	2.6646
0.3660	3.4271	3.2688	3.2778	3.2488	2.5738	2.6106	2.6706
0.3720	3.4621	3.2928	3.3038	3.2718	2.5738	2.6226	2.6746
0.3780	3.5011	3.3188	3.3328	3.2968	2.5858	2.6276	2.6816
0.3840	3.5431	3.3448	3.3628	3.3208	2.5858	2.6326	2.6866
0.3900	3.5881	3.3718	3.3918	3.3468	2.5998	2.6396	2.6916
0.3960	3.6341	3.3988	3.4218	3.3728	2.6058	2.6446	2.6986
0.4020	3.6831	3.4268	3.4538	3.4008	2.6118	2.6506	2.7046
0.4080	3.7371	3.4558	3.4878	3.4288	2.6188	2.6566	2.7106
0.4140	3.7941	3.4858	3.5228	3.4578	2.6258	2.6626	2.7176
0.4200	3.8561	3.5168	3.5568	3.4858	2.6318	2.6626	2.7236
0.4260	3.9231	3.5488	3.5948	3.5158	2.6388	2.6746	2.7296
0.4320	3.9981	3.5808	3.6338	3.5468	2.6388	2.6816	2.7366
0.4380	4.0801	3.6178	3.6748	3.5778	2.6458	2.6806	2.7436
0.4440	4.1741	3.6508	3.7168	3.6098	2.6598	2.6866	2.7506
0.4500	4.2881	3.6868	3.7598	3.6438	2.6668	2.7006	2.7566
0.4560	4.4321	3.7228	3.8058	3.6808	2.6738	2.7066	2.7646
0.4620	4.6271	3.7618	3.8548	3.7158	2.6818	2.7126	2.7716
0.4680	4.9491	3.8018	3.9048	3.7518	2.6888	2.7196	2.7776
0.4740	5.8701	3.8428	3.9608	3.7898	2.6958	2.7266	2.7846
0.4800	6.8501	3.8878	4.0208	3.8318	2.7048	2.7336	2.7936
0.4860	7.1931	3.9348	4.0878	3.8738	2.7138	2.7396	2.8006
0.4920	7.3471	3.9868	4.1638	3.9188	2.7208	2.7466	2.8076
0.4980	7.4541	4.0388	4.2488	3.9678	2.7288	2.7536	2.8156

0.5040	7.5301	4.0978	4.3478	4.0178	2.7378	2.7616	2.8226
0.5100	7.5831	4.1618	4.4658	4.0738	2.7468	2.7676	2.8306
0.5160	7.6291	4.2368	4.6238	4.1338	2.7558	2.7766	2.8396
0.5220	7.6661	4.3168	4.8538	4.1988	2.7658	2.7836	2.8476
0.5280	7.6971	4.4098	5.2588	4.2718	2.7738	2.7906	2.8546
0.5340	7.7201	4.5178	6.2758	4.3568	2.7808	2.7986	2.8636
0.5400	7.7411	4.6548	6.7808	4.4538	2.7918	2.8066	2.8746
0.5460	7.7621	4.8418	6.9848	4.5768	2.8008	2.8146	2.8836
0.5520	7.7811	5.1488	7.1058	4.7368	2.8098	2.8226	2.8916
0.5580	7.7981	5.9368	7.2228	4.9718	2.8208	2.8296	2.9006
0.5640	7.8141	6.6938	7.3078	5.4168	2.8308	2.8386	2.9106
0.5700	7.8271	7.0018	7.3908	6.3858	2.8408	2.8476	2.9196
0.5760	7.8431	7.1908	7.4568	6.8858	2.8508	2.8556	2.9296
0.5820	7.8581	7.3248	7.5108	7.1158	2.8618	2.8656	2.9406
0.5880	7.8721	7.4338	7.5568	7.2528	2.8728	2.8766	2.9526
0.5940	7.8931	7.5308	7.5958	7.3618	2.8838	2.8856	2.9636
0.6000	7.9031	7.6068	7.6308	7.4518	2.8948	2.8936	2.9746
0.6060	7.9211	7.6768	7.6698	7.5168	2.9048	2.9046	2.9846
0.6120	7.9211	7.7388	7.6978	7.5678	2.9158	2.9126	2.9956
0.6180	7.9501	7.7388	7.7118	7.6108	2.9278	2.9236	3.0066
0.6240	7.9661	7.8198	7.7358	7.6458	2.9388	2.9336	3.0186
0.6300	7.9861	7.8798	7.7528	7.6768	2.9508	2.9436	3.0286
0.6360	8.0041	7.9268	7.7718	7.6998	2.9628	2.9526	3.0396
0.6420	8.0251	7.9748	7.7918	7.7208	2.9758	2.9636	3.0506
0.6480	8.0261	8.0128	7.8158	7.7388	2.9898	2.9756	3.0636
0.6540	8.0811	8.0508	7.8278	7.7578	3.0018	2.9856	3.0756
0.6600	8.1021	8.0878	7.8438	7.7738	3.0158	2.9976	3.0896
0.6660	8.1301	8.1248	7.8628	7.7898	3.0228	3.0076	3.1016
0.6720	8.1581	8.1618	7.8828	7.8058	3.0368	3.0196	3.1156
0.6780	8.1911	8.1918	7.9008	7.8198	3.0568	3.0326	3.1296
0.6840	8.2311	8.2228	7.9178	7.8348	3.0708	3.0446	3.1446
0.6900	8.2871	8.2498	7.9368	7.8498	3.0868	3.0496	3.1516
0.6960	8.3531	8.2798	7.9688	7.8648	3.0998	3.0616	3.1686
0.7020	8.4471	8.3038	7.9838	7.8808	3.1168	3.0736	3.1826
0.7080	8.6051	8.3268	8.0038	7.8958	3.1328	3.0896	3.1986
0.7140	8.8801	8.3428	8.0238	7.9118	3.1488	3.1096	3.2176
0.7200	9.2181	8.3648	8.0448	7.9258	3.1638	3.1156	3.2296
0.7260	9.5281	8.3858	8.0728	7.9428	3.1818	3.1376	3.2486
0.7320	9.7651	8.4098	8.0968	7.9598	3.1988	3.1446	3.2626
0.7380	9.9571	8.4348	8.1288	7.9788	3.2108	3.1646	3.2806
0.7440	10.0991	8.4558	8.1598	7.9978	3.2278	3.1756	3.3016
0.7500	10.2161	8.4808	8.2018	8.0208	3.2468	3.1916	3.3166
0.7560	10.3151	8.5078	8.2538	8.0418	3.2658	3.2086	3.3346
0.7620	10.3981	8.5348	8.3228	8.0678	3.2838	3.2256	3.3576
0.7680	10.4691	8.5628	8.3788	8.0938	3.3098	3.2466	3.3776
0.7740	10.5331	8.5958	8.4748	8.1238	3.3298	3.2606	3.3956
0.7800	10.5901	8.6308	8.6318	8.1598	3.3488	3.2846	3.4196
0.7860	10.6401	8.6678	8.8798	8.1988	3.3718	3.3036	3.4396
0.7920	10.6871	8.7118	9.2018	8.2468	3.3928	3.3176	3.4626
0.7980	10.7301	8.7658	9.4928	8.3058	3.4078	3.3416	3.4846
0.8040	10.7671	8.8178	9.7318	8.3928	3.4368	3.3576	3.5066

0.8100	10.8031	8.8958	9.9218	8.4998	3.4598	3.3846	3.5336
0.8160	10.8341	8.9968	10.0748	8.6648	3.4838	3.4056	3.5576
0.8220	10.8641	9.1298	10.1978	8.9318	3.5078	3.4276	3.5836
0.8280	10.8941	9.2998	10.2988	9.2438	3.5338	3.4526	3.6116
0.8340	10.9201	9.4948	10.3838	9.5148	3.5598	3.4746	3.6386
0.8400	10.9461	9.6878	10.4618	9.7408	3.5808	3.5006	3.6656
0.8460	10.9711	9.8588	10.5298	9.9288	3.6098	3.5266	3.6976
0.8520		10.0028	10.5888	10.0758	3.6378	3.5526	3.7286
0.8580		10.1298	10.6428	10.2008	3.6718	3.5796	3.7596
0.8640		10.2368	10.6888	10.3038	3.7008	3.6076	3.7956
0.8700		10.3288	10.7308	10.3928	3.7328	3.6356	3.8306
0.8760		10.4098	10.7708	10.4668	3.7638	3.6666	3.8706
0.8820		10.4878	10.8088	10.5318	3.7968	3.6966	3.9096
0.8880		10.5508	10.8428	10.5888	3.8318	3.7316	3.9516
0.8940		10.6078	10.8748	10.6418	3.8678	3.7646	3.9946
0.9000		10.6598	10.9048	10.6898	3.9048	3.7996	4.0396
0.9060		10.7018	10.9328	10.7348	3.9428	3.8356	4.0936
0.9120		10.7478	10.9608	10.7748	3.9868	3.8756	4.1446
0.9180		10.7878	10.9848	10.8128	4.0278	3.9176	4.2056
0.9240		10.8228	11.0098	10.8468	4.0778	3.9606	4.2736
0.9300		10.8568		10.8788	4.1278	4.0066	4.3516
0.9360		10.8908		10.9088	4.1848	4.0566	4.4376
0.9420		10.9218		10.9358	4.2468	4.1116	4.5356
0.9480		10.9488		10.9638	4.3128	4.1706	4.6796
0.9540		10.9738		10.9878	4.3918	4.2366	4.8606
0.9600		10.9988		11.0128	4.4858	4.3096	5.1456
0.9660		11.0228			4.5948	4.3926	5.7406
0.9720					4.7358	4.4906	6.5756
0.9780					4.9288	4.6086	6.9376
0.9840					5.2418	4.7646	7.0556
0.9900					5.8798	4.9866	7.1096
0.9960					6.7288	5.4056	7.1606
1.0020					7.0278	6.3426	7.1836
1.0080					7.1838	7.0696	7.2196
1.0140					7.2728	7.2686	7.2426
1.0200					7.3388	7.3636	7.2696
1.0260					7.3888	7.4206	7.2886
1.0320					7.4338	7.4636	7.3056
1.0380					7.4648	7.4966	7.3226
1.0440					7.4958	7.5256	7.3406
1.0500					7.5178	7.5516	7.3586
1.0560					7.5418	7.5736	7.3716
1.0620					7.5688	7.5936	7.3886
1.0680					7.5858	7.6106	7.4036
1.0740					7.6028	7.6256	7.4206
1.0800					7.6188	7.6446	7.4336
1.0860					7.6308	7.6616	7.4486
1.0920					7.6448	7.6746	7.4496
1.0980					7.6598	7.6886	7.4806
1.1040					7.6738	7.7036	7.4946
1.1100					7.6838	7.7166	7.5116

1.1160					7.6888	7.7306	7.5276
1.1220					7.7018	7.7416	7.5466
1.1280					7.7188	7.7566	7.5616
1.1340					7.7348	7.7696	7.5806
1.1400					7.7478	7.7846	7.6016
1.1460					7.7598	7.8026	7.6176
1.1520					7.7618	7.8186	7.6406
1.1580					7.7838	7.8326	7.6606
1.1640					7.8028	7.8526	7.6886
1.1700					7.8278	7.8536	7.7186
1.1760					7.8438	7.8746	7.7466
1.1820					7.8698	7.8936	7.7796
1.1880					7.8918	7.9196	7.8176
1.1940					7.9188	7.9546	7.8786
1.2000					7.9468	7.9736	7.9516
1.2060					7.9668	8.0046	8.0406
1.2120					8.0108	8.0406	8.2086
1.2180					8.0488	8.0926	8.5176
1.2240					8.1018	8.1456	8.8906
1.2300					8.1728	8.2126	9.1886
1.2360					8.2488	8.3116	9.3876
1.2420					8.3808	8.4546	9.5976
1.2480					8.5878	8.6836	9.7736
1.2540					8.8908	8.9356	9.9126
1.2600					9.1558	9.1906	10.0376
1.2660					9.3788	9.3376	10.1246
1.2720					9.5718	9.5326	10.2096
1.2780					9.7308	9.7026	10.2606
1.2840					9.8688	9.8056	10.2866
1.2900					9.9638	9.9106	10.3716
1.2960					10.0608	10.0466	10.4426
1.3020					10.1468	10.1366	10.4956
1.3080					10.2228	10.1886	10.5336
1.3140					10.2918	10.2806	10.5986
1.3200					10.3528	10.3506	10.6336
1.3260					10.4028	10.4156	10.6706
1.3320					10.4548	10.4786	10.7066
1.3380					10.4998	10.5186	10.7456
1.3440					10.5378	10.5666	10.7726
1.3500					10.5758	10.6206	10.8016
1.3560					10.6098	10.6556	10.8266
1.3620					10.6428	10.7016	10.8566
1.3680					10.6718	10.7356	10.8786
1.3740					10.6998	10.7676	10.9026
1.3800					10.7278	10.7926	10.9206
1.3860					10.7528	10.8236	10.9456
1.3920					10.7778	10.8476	10.9676
1.3980					10.7998	10.8726	10.9866
1.4040					10.8208	10.8996	11.0026
1.4100					10.8418	10.9246	11.0236
1.4160					10.8598	10.9436	11.0396

1.4220					10.8808	10.9666	11.0566
1.4280					10.8998	10.9876	11.0736
1.4340					10.9178	11.0066	
1.4400					10.9348	11.0246	
1.4460					10.9508	11.0396	
1.4520					10.9668	11.0566	
1.4580						11.0756	

Table S3. Data obtained from potentiometric measurements during studies of glycolic acid/spermine binary systems.

**V [ml]**	**pH**
	**GA/Spm**
0.0000	2.2708
0.0060	2.2708
0.0120	2.2768
0.0180	2.2808
0.0240	2.2848
0.0300	2.2898
0.0360	2.2938
0.0420	2.2978
0.0480	2.3018
0.0540	2.3058
0.0600	2.3068
0.0660	2.3138
0.0720	2.3188
0.0780	2.3228
0.0840	2.3278
0.0900	2.3328
0.0960	2.3368
0.1020	2.3418
0.1080	2.3468
0.1140	2.3468
0.1200	2.3558
0.1260	2.3558
0.1320	2.3648
0.1380	2.3698
0.1440	2.3738
0.1500	2.3798
0.1560	2.3838
0.1620	2.3888
0.1680	2.3938
0.1740	2.3988
0.1800	2.4038
0.1860	2.4098
0.1920	2.4148
0.1980	2.4198
0.2040	2.4248
0.2100	2.4248
0.2160	2.4358
0.2220	2.4408

0.2280	2.4458
0.2340	2.4518
0.2400	2.4578
0.2460	2.4628
0.2520	2.4688
0.2580	2.4748
0.2640	2.4808
0.2700	2.4858
0.2760	2.4928
0.2820	2.4988
0.2880	2.5048
0.2940	2.5108
0.3000	2.5168
0.3060	2.5238
0.3120	2.5288
0.3180	2.5358
0.3240	2.5428
0.3300	2.5498
0.3360	2.5568
0.3420	2.5638
0.3480	2.5708
0.3540	2.5778
0.3600	2.5848
0.3660	2.5918
0.3720	2.5988
0.3780	2.6058
0.3840	2.6068
0.3900	2.6208
0.3960	2.6298
0.4020	2.6378
0.4080	2.6458
0.4140	2.6538
0.4200	2.6648
0.4260	2.6728
0.4320	2.6818
0.4380	2.6908
0.4440	2.7008
0.4500	2.7078
0.4560	2.7178
0.4620	2.7268
0.4680	2.7378
0.4740	2.7458
0.4800	2.7558
0.4860	2.7668
0.4920	2.7768
0.4980	2.7878
0.5040	2.7998
0.5100	2.8098
0.5160	2.8218
0.5220	2.8338
0.5280	2.8458

0.5340	2.8568
0.5400	2.8698
0.5460	2.8828
0.5520	2.8948
0.5580	2.9088
0.5640	2.9228
0.5700	2.9368
0.5760	2.9508
0.5820	2.9658
0.5880	2.9798
0.5940	2.9958
0.6000	3.0108
0.6060	3.0268
0.6120	3.0438
0.6180	3.0598
0.6240	3.0768
0.6300	3.0948
0.6360	3.1128
0.6420	3.1308
0.6480	3.1498
0.6540	3.1688
0.6600	3.1898
0.6660	3.2088
0.6720	3.2298
0.6780	3.2508
0.6840	3.2728
0.6900	3.2948
0.6960	3.3168
0.7020	3.3408
0.7080	3.3648
0.7140	3.3888
0.7200	3.4138
0.7260	3.4398
0.7320	3.4658
0.7380	3.4928
0.7440	3.5218
0.7500	3.5508
0.7560	3.5778
0.7620	3.6088
0.7680	3.6398
0.7740	3.6718
0.7800	3.7058
0.7860	3.7398
0.7920	3.7748
0.7980	3.8118
0.8040	3.8498
0.8100	3.8898
0.8160	3.9298
0.8220	3.9748
0.8280	4.0198
0.8340	4.0698

0.8400	4.1218
0.8460	4.1798
0.8520	4.2408
0.8580	4.3108
0.8640	4.3898
0.8700	4.4828
0.8760	4.5898
0.8820	4.7278
0.8880	4.7298
0.8940	5.2188
0.9000	5.8348
0.9060	6.4448
0.9120	6.7618
0.9180	6.9768
0.9240	7.1498
0.9300	7.2818
0.9360	7.2838
0.9420	7.4798
0.9480	7.5598
0.9540	7.6308
0.9600	7.6988
0.9660	7.7578
0.9720	7.8128
0.9780	7.8628
0.9840	7.9128
0.9900	7.9608
0.9960	8.0038
1.0020	8.0478
1.0080	8.0478
1.0140	8.1268
1.0200	8.1658
1.0260	8.2058
1.0320	8.2438
1.0380	8.2788
1.0440	8.3158
1.0500	8.3518
1.0560	8.3528
1.0620	8.4228
1.0680	8.4568
1.0740	8.4908
1.0800	8.5238
1.0860	8.5248
1.0920	8.5888
1.0980	8.6218
1.1040	8.6548
1.1100	8.6858
1.1160	8.7178
1.1220	8.7188
1.1280	8.7818
1.1340	8.8148
1.1400	8.8158

1.1460	8.8768
1.1520	8.8788
1.1580	8.9428
1.1640	8.9738
1.1700	9.0048
1.1760	9.0348
1.1820	9.0678
1.1880	9.1008
1.1940	9.1298
1.2000	9.1318
1.2060	9.1948
1.2120	9.2258
1.2180	9.2268
1.2240	9.2898
1.2300	9.3208
1.2360	9.3538
1.2420	9.3558
1.2480	9.4198
1.2540	9.4508
1.2600	9.4828
1.2660	9.5148
1.2720	9.5478
1.2780	9.5788
1.2840	9.6108
1.2900	9.6418
1.2960	9.6728
1.3020	9.6728
1.3080	9.7338
1.3140	9.7598
1.3200	9.7608
1.3260	9.8188
1.3320	9.8448
1.3380	9.8738
1.3440	9.8998
1.3500	9.9018
1.3560	9.9488
1.3620	9.9798
1.3680	10.004
1.3740	10.029
1.3800	10.053
1.3860	10.077
1.3920	10.078
1.3980	10.124
1.4040	10.149
1.4100	10.170
1.4160	10.171
1.4220	10.216
1.4280	10.218
1.4340	10.262
1.4400	10.282
1.4460	10.305

1.4520	10.324
1.4580	10.345
1.4640	10.364
1.4700	10.381
1.4760	10.403
1.4820	10.421
1.4880	10.438
1.4940	10.457
1.5000	10.476
1.5060	10.494
1.5120	10.511
1.5180	10.529
1.5240	10.546
1.5300	10.561
1.5360	10.579
1.5420	10.594
1.5480	10.610
1.5540	10.626
1.5600	10.641
1.5660	10.657
1.5720	10.658
1.5780	10.688
1.5840	10.702
1.5900	10.716
1.5960	10.731
1.6020	10.745
1.6080	10.759
1.6140	10.773
1.6200	10.786
1.6260	10.799
1.6320	10.811
1.6380	10.825
1.6440	10.836
1.6500	10.848
1.6560	10.860
1.6620	10.861
1.6680	10.884
1.6740	10.896
1.6800	10.907

Table S4. Data obtained from potentiometric measurements during studies of lanthanide ion/tartaric acid/spermine ternary systems.

**V [ml]**	**pH**						
	**La(III)**	**Nd(III)**	**Eu(III)**	**Gd(III)**	**Tb(III)**	**Ho(III)**	**Lu(III)**
0.0000	2.2690	2.2738	2.2769	2.2953	2.2562	2.2745	2.2809
0.0060	2.2700	2.2758	2.2789	2.2963	2.2582	2.2775	2.2829
0.0120	2.2730	2.2788	2.2809	2.3003	2.2612	2.2815	2.2869
0.0180	2.2770	2.2828	2.2839	2.3033	2.2652	2.2855	2.2899

0.0240	2.2790	2.2858	2.2859	2.3063	2.2682	2.2885	2.2929
0.0300	2.2830	2.2888	2.2899	2.3103	2.2722	2.2925	2.2969
0.0360	2.2860	2.2928	2.2919	2.3133	2.2752	2.2955	2.3009
0.0420	2.2900	2.2968	2.2949	2.3173	2.2792	2.2995	2.3049
0.0480	2.2930	2.2998	2.2989	2.3203	2.2822	2.3045	2.3069
0.0540	2.2980	2.3038	2.3019	2.3243	2.2862	2.3075	2.3129
0.0600	2.3020	2.3068	2.3049	2.3283	2.2892	2.3115	2.3159
0.0660	2.3060	2.3108	2.3079	2.3313	2.2932	2.3145	2.3199
0.0720	2.3090	2.3158	2.3119	2.3353	2.2982	2.3205	2.3239
0.0780	2.3140	2.3198	2.3149	2.3403	2.3012	2.3235	2.3279
0.0840	2.3180	2.3228	2.3189	2.3443	2.3052	2.3275	2.3319
0.0900	2.3210	2.3258	2.3229	2.3483	2.3092	2.3325	2.3359
0.0960	2.3260	2.3298	2.3269	2.3523	2.3122	2.3355	2.3399
0.1020	2.3300	2.3358	2.3299	2.3563	2.3172	2.3405	2.3439
0.1080	2.3340	2.3388	2.3339	2.3603	2.3212	2.3445	2.3479
0.1140	2.3390	2.3418	2.3379	2.3653	2.3242	2.3485	2.3529
0.1200	2.3420	2.3478	2.3409	2.3683	2.3302	2.3525	2.3559
0.1260	2.3460	2.3508	2.3459	2.3733	2.3332	2.3565	2.3609
0.1320	2.3510	2.3548	2.3499	2.3773	2.3372	2.3615	2.3649
0.1380	2.3560	2.3588	2.3529	2.3823	2.3402	2.3615	2.3699
0.1440	2.3600	2.3638	2.3579	2.3863	2.3452	2.3705	2.3739
0.1500	2.3630	2.3688	2.3619	2.3913	2.3512	2.3745	2.3789
0.1560	2.3690	2.3728	2.3659	2.3953	2.3542	2.3795	2.3839
0.1620	2.3740	2.3778	2.3709	2.4003	2.3592	2.3845	2.3879
0.1680	2.3780	2.3818	2.3749	2.4043	2.3632	2.3845	2.3919
0.1740	2.3830	2.3868	2.3789	2.4083	2.3662	2.3935	2.3959
0.1800	2.3880	2.3898	2.3839	2.4133	2.3712	2.3975	2.4019
0.1860	2.3920	2.3958	2.3869	2.4173	2.3752	2.4025	2.4049
0.1920	2.3960	2.3998	2.3919	2.4223	2.3792	2.4055	2.4099
0.1980	2.4000	2.4038	2.3959	2.4263	2.3842	2.4105	2.4139
0.2040	2.4060	2.4088	2.4009	2.4313	2.3872	2.4145	2.4169
0.2100	2.4110	2.4128	2.4049	2.4353	2.3932	2.4195	2.4229
0.2160	2.4150	2.4168	2.4089	2.4403	2.3982	2.4235	2.4279
0.2220	2.4200	2.4218	2.4139	2.4443	2.4012	2.4285	2.4309
0.2280	2.4240	2.4268	2.4169	2.4493	2.4072	2.4335	2.4369
0.2340	2.4290	2.4308	2.4229	2.4543	2.4112	2.4385	2.4399
0.2400	2.4340	2.4358	2.4269	2.4593	2.4152	2.4425	2.4449
0.2460	2.4380	2.4418	2.4309	2.4633	2.4212	2.4475	2.4499
0.2520	2.4430	2.4468	2.4359	2.4683	2.4242	2.4535	2.4539
0.2580	2.4480	2.4518	2.4399	2.4733	2.4292	2.4585	2.4589
0.2640	2.4530	2.4558	2.4449	2.4783	2.4352	2.4615	2.4639
0.2700	2.4580	2.4608	2.4489	2.4823	2.4382	2.4665	2.4699
0.2760	2.4640	2.4658	2.4539	2.4873	2.4442	2.4725	2.4729
0.2820	2.4690	2.4718	2.4589	2.4923	2.4492	2.4775	2.4779
0.2880	2.4740	2.4768	2.4629	2.4983	2.4542	2.4825	2.4839
0.2940	2.4790	2.4798	2.4689	2.5033	2.4572	2.4875	2.4889
0.3000	2.4850	2.4858	2.4739	2.5083	2.4632	2.4935	2.4939
0.3060	2.4900	2.4908	2.4779	2.5143	2.4682	2.4975	2.4989
0.3120	2.4950	2.4958	2.4829	2.5193	2.4722	2.5035	2.5049
0.3180	2.5010	2.5018	2.4879	2.5243	2.4792	2.5075	2.5099
0.3240	2.5060	2.5068	2.4929	2.5293	2.4822	2.5135	2.5149

0.3300	2.5110	2.5118	2.4989	2.5343	2.4872	2.5195	2.5199
0.3360	2.5170	2.5178	2.5039	2.5403	2.4932	2.5245	2.5249
0.3420	2.5240	2.5238	2.5089	2.5453	2.4982	2.5295	2.5309
0.3480	2.5290	2.5288	2.5129	2.5503	2.5032	2.5355	2.5359
0.3540	2.5340	2.5348	2.5189	2.5573	2.5082	2.5415	2.5409
0.3600	2.5390	2.5398	2.5229	2.5623	2.5132	2.5475	2.5459
0.3660	2.5450	2.5458	2.5289	2.5683	2.5192	2.5525	2.5519
0.3720	2.5510	2.5508	2.5339	2.5733	2.5242	2.5575	2.5569
0.3780	2.5570	2.5578	2.5389	2.5783	2.5292	2.5635	2.5629
0.3840	2.5620	2.5628	2.5449	2.5853	2.5352	2.5695	2.5689
0.3900	2.5690	2.5698	2.5509	2.5913	2.5412	2.5755	2.5689
0.3960	2.5760	2.5758	2.5569	2.5973	2.5472	2.5825	2.5809
0.4020	2.5820	2.5818	2.5619	2.6033	2.5522	2.5875	2.5869
0.4080	2.5890	2.5868	2.5679	2.6103	2.5582	2.5935	2.5919
0.4140	2.5960	2.5928	2.5729	2.6153	2.5632	2.5995	2.5979
0.4200	2.6010	2.5998	2.5789	2.6223	2.5682	2.6065	2.6039
0.4260	2.6080	2.6068	2.5849	2.6293	2.5752	2.6125	2.6109
0.4320	2.6140	2.6128	2.5899	2.6353	2.5802	2.6195	2.6159
0.4380	2.6210	2.6198	2.5959	2.6413	2.5852	2.6245	2.6229
0.4440	2.6290	2.6268	2.6019	2.6483	2.5932	2.6315	2.6279
0.4500	2.6350	2.6328	2.6079	2.6553	2.5982	2.6385	2.6349
0.4560	2.6430	2.6388	2.6149	2.6613	2.6052	2.6455	2.6419
0.4620	2.6500	2.6458	2.6209	2.6683	2.6102	2.6525	2.6479
0.4680	2.6570	2.6528	2.6259	2.6753	2.6172	2.6595	2.6549
0.4740	2.6640	2.6598	2.6329	2.6813	2.6222	2.6665	2.6619
0.4800	2.6710	2.6678	2.6399	2.6883	2.6282	2.6735	2.6689
0.4860	2.6800	2.6738	2.6459	2.6963	2.6352	2.6805	2.6749
0.4920	2.6870	2.6818	2.6529	2.7043	2.6422	2.6875	2.6829
0.4980	2.6950	2.6898	2.6599	2.7113	2.6492	2.6955	2.6899
0.5040	2.7050	2.6968	2.6669	2.7193	2.6562	2.7035	2.6969
0.5100	2.7130	2.7038	2.6739	2.7273	2.6612	2.7115	2.7029
0.5160	2.7200	2.7148	2.6809	2.7343	2.6692	2.7185	2.7129
0.5220	2.7290	2.7218	2.6879	2.7433	2.6772	2.7275	2.7209
0.5280	2.7380	2.7288	2.6949	2.7503	2.6832	2.7345	2.7279
0.5340	2.7450	2.7378	2.7019	2.7583	2.6902	2.7435	2.7369
0.5400	2.7530	2.7458	2.7089	2.7683	2.6982	2.7495	2.7439
0.5460	2.7630	2.7538	2.7159	2.7763	2.7042	2.7575	2.7509
0.5520	2.7710	2.7618	2.7229	2.7853	2.7102	2.7655	2.7599
0.5580	2.7800	2.7718	2.7319	2.7933	2.7192	2.7755	2.7669
0.5640	2.7890	2.7788	2.7389	2.8023	2.7262	2.7845	2.7749
0.5700	2.7980	2.7868	2.7479	2.8113	2.7342	2.7925	2.7839
0.5760	2.8080	2.7978	2.7549	2.8203	2.7412	2.8005	2.7919
0.5820	2.8170	2.8058	2.7619	2.8283	2.7512	2.8115	2.8009
0.5880	2.8280	2.8158	2.7709	2.8373	2.7592	2.8205	2.8099
0.5940	2.8360	2.8238	2.7789	2.8473	2.7682	2.8295	2.8189
0.6000	2.8490	2.8338	2.7869	2.8573	2.7722	2.8375	2.8279
0.6060	2.8570	2.8438	2.7959	2.8653	2.7812	2.8475	2.8359
0.6120	2.8680	2.8528	2.8039	2.8763	2.7912	2.8575	2.8439
0.6180	2.8780	2.8628	2.8119	2.8863	2.7992	2.8655	2.8539
0.6240	2.8910	2.8728	2.8209	2.8963	2.8062	2.8765	2.8629
0.6300	2.9010	2.8828	2.8299	2.9063	2.8152	2.8865	2.8719

0.6360	2.9120	2.8928	2.8379	2.9163	2.8242	2.8965	2.8819
0.6420	2.9230	2.9038	2.8469	2.9263	2.8332	2.9075	2.8919
0.6480	2.9350	2.9138	2.8559	2.9383	2.8412	2.9175	2.9019
0.6540	2.9470	2.9248	2.8649	2.9493	2.8502	2.9285	2.9129
0.6600	2.9600	2.9348	2.8749	2.9603	2.8592	2.9385	2.9229
0.6660	2.9720	2.9468	2.8839	2.9723	2.8692	2.9495	2.9329
0.6720	2.9840	2.9588	2.8929	2.9833	2.8782	2.9605	2.9439
0.6780	2.9950	2.9688	2.9039	2.9953	2.8872	2.9725	2.9549
0.6840	3.0100	2.9808	2.9129	3.0073	2.8972	2.9845	2.9649
0.6900	3.0230	2.9938	2.9229	3.0193	2.9062	2.9955	2.9769
0.6960	3.0370	3.0058	2.9339	3.0313	2.9152	3.0075	2.9879
0.7020	3.0510	3.0178	2.9429	3.0443	2.9262	3.0195	2.9999
0.7080	3.0650	3.0308	2.9539	3.0563	2.9362	3.0315	3.0109
0.7140	3.0790	3.0428	2.9639	3.0683	2.9452	3.0445	3.0229
0.7200	3.0930	3.0568	2.9749	3.0823	2.9552	3.0585	3.0349
0.7260	3.1080	3.0698	2.9849	3.0943	2.9662	3.0705	3.0469
0.7320	3.1230	3.0828	2.9959	3.1093	2.9762	3.0825	3.0599
0.7380	3.1390	3.0978	3.0069	3.1233	2.9872	3.0975	3.0719
0.7440	3.1550	3.1118	3.0189	3.1373	2.9972	3.1115	3.0859
0.7500	3.1700	3.1258	3.0309	3.1523	3.0082	3.1245	3.0999
0.7560	3.1880	3.1418	3.0419	3.1673	3.0202	3.1405	3.1139
0.7620	3.2050	3.1558	3.0539	3.1823	3.0292	3.1545	3.1269
0.7680	3.2200	3.1718	3.0669	3.1983	3.0422	3.1685	3.1419
0.7740	3.2410	3.1888	3.0789	3.2153	3.0552	3.1855	3.1569
0.7800	3.2600	3.2058	3.0919	3.2313	3.0672	3.2015	3.1709
0.7860	3.2790	3.2218	3.1039	3.2493	3.0772	3.2185	3.1869
0.7920	3.2990	3.2388	3.1179	3.2653	3.0922	3.2345	3.2019
0.7980	3.3200	3.2568	3.1309	3.2843	3.1042	3.2525	3.2179
0.8040	3.3410	3.2758	3.1449	3.3033	3.1172	3.2705	3.2359
0.8100	3.3630	3.2938	3.1589	3.3223	3.1302	3.2875	3.2519
0.8160	3.3850	3.3128	3.1729	3.3413	3.1432	3.3065	3.2699
0.8220	3.4080	3.3338	3.1869	3.3613	3.1562	3.3255	3.2879
0.8280	3.4310	3.3538	3.2009	3.3813	3.1692	3.3445	3.3049
0.8340	3.4540	3.3748	3.2169	3.4033	3.1832	3.3655	3.3249
0.8400	3.4800	3.3958	3.2309	3.4243	3.1972	3.3855	3.3449
0.8460	3.5060	3.4188	3.2459	3.4463	3.2112	3.4065	3.3649
0.8520	3.5330	3.4418	3.2619	3.4693	3.2262	3.4285	3.3839
0.8580	3.5620	3.4668	3.2769	3.4943	3.2412	3.4515	3.4069
0.8640	3.5910	3.4898	3.2949	3.5193	3.2572	3.4745	3.4289
0.8700	3.6210	3.5168	3.3109	3.5453	3.2732	3.4985	3.4509
0.8760	3.6520	3.5438	3.3269	3.5723	3.2872	3.5235	3.4739
0.8820	3.6840	3.5728	3.3449	3.6003	3.3022	3.5495	3.4989
0.8880	3.7190	3.6008	3.3629	3.6313	3.3202	3.5775	3.5239
0.8940	3.7540	3.6328	3.3819	3.6623	3.3362	3.6065	3.5499
0.9000	3.7920	3.6638	3.4009	3.6943	3.3532	3.6355	3.5779
0.9060	3.8320	3.6968	3.4199	3.7273	3.3702	3.6665	3.6059
0.9120	3.8740	3.7308	3.4399	3.7633	3.3872	3.6995	3.6349
0.9180	3.9190	3.7698	3.4599	3.8003	3.4062	3.7335	3.6649
0.9240	3.9690	3.8088	3.4809	3.8423	3.4252	3.7705	3.6969
0.9300	4.0210	3.8528	3.5039	3.8863	3.4442	3.8085	3.7279
0.9360	4.0790	3.8978	3.5259	3.9353	3.4642	3.8495	3.7629

0.9420	4.1440	3.9488	3.5489	3.9873	3.4832	3.8925	3.7959
0.9480	4.2150	4.0028	3.5739	4.0433	3.5052	3.9375	3.8329
0.9540	4.2960	4.0628	3.6019	4.1043	3.5272	3.9865	3.8709
0.9600	4.3890	4.1288	3.6279	4.1713	3.5502	4.0375	3.9089
0.9660	4.5020	4.2028	3.6539	4.2463	3.5722	4.0935	3.9489
0.9720	4.6380	4.2888	3.6829	4.3303	3.5982	4.1515	3.9899
0.9780	4.8160	4.3858	3.7109	4.4233	3.6222	4.2145	4.0299
0.9840	5.0600	4.5078	3.7409	4.5253	3.6482	4.2805	4.0729
0.9900	5.3940	4.6528	3.7729	4.6243	3.6742	4.3495	4.1169
0.9960	5.7530	4.8218	3.8069	4.7193	3.7002	4.4185	4.1619
1.0020	5.9590	4.9968	3.8419	4.8033	3.7292	4.4875	4.2079
1.0080	6.1750	5.1428	3.8789	4.8833	3.7582	4.5555	4.2549
1.0140	6.3050	5.2558	3.9179	4.9533	3.7892	4.6235	4.3029
1.0200	6.3980	5.3478	3.9609	5.0203	3.8212	4.6905	4.3559
1.0260	6.4690	5.4248	4.0029	5.0813	3.8552	4.7565	4.4099
1.0320	6.5300	5.4948	4.0509	5.1423	3.8892	4.8225	4.4679
1.0380	6.5840	5.5598	4.1019	5.2023	3.9272	4.8855	4.5269
1.0440	6.6290	5.6228	4.1569	5.2633	3.9652	4.9535	4.5919
1.0500	6.6780	5.6848	4.2159	5.3253	4.0072	5.0225	4.6599
1.0560	6.7200	5.7468	4.2769	5.3873	4.0482	5.0905	4.7299
1.0620	6.7640	5.8078	4.3449	5.4523	4.0952	5.1635	4.8079
1.0680	6.8010	5.8718	4.4179	5.5203	4.1422	5.2395	4.8919
1.0740	6.8340	5.9408	4.4949	5.5963	4.1912	5.3235	4.9839
1.0800	6.8730	6.0098	4.5699	5.6753	4.2432	5.4125	5.0869
1.0860	6.9090	6.0848	4.6489	5.7593	4.2982	5.5085	5.2019
1.0920	6.9400	6.1668	4.7239	5.8533	4.3542	5.6165	5.3369
1.0980	6.9780	6.2568	4.7909	5.9583	4.4142	5.7395	5.4939
1.1040	7.0110	6.3538	4.8559	6.0743	4.4752	5.8745	5.6839
1.1100	7.0440	6.4628	4.9189	6.2093	4.5382	6.0205	5.9029
1.1160	7.0800	6.5748	4.9789	6.3543	4.5962	6.1695	6.1289
1.1220	7.1190	6.6938	5.0369	6.5103	4.6552	6.3125	6.3049
1.1280	7.1670	6.8158	5.0939	6.6643	4.7132	6.4435	6.4339
1.1340	7.2070	6.9338	5.1519	6.8123	4.7712	6.5505	6.5399
1.1400	7.2560	7.0358	5.2109	6.9453	4.8272	6.6705	6.4599
1.1460	7.3000	7.1338	5.2709	7.0573	4.8832	6.7815	6.5969
1.1520	7.3440	7.2218	5.3349	7.1623	4.9382	6.8845	6.7079
1.1580	7.3930	7.3048	5.3979	7.2533	4.9922	6.9715	6.8079
1.1640	7.4390	7.3768	5.4669	7.3353	5.0492	7.0615	6.8999
1.1700	7.4870	7.4458	5.5379	7.4093	5.1062	7.1475	6.9799
1.1760	7.5300	7.5078	5.6169	7.4773	5.1662	7.2295	7.0609
1.1820	7.5770	7.5658	5.7009	7.5393	5.2232	7.3075	7.1409
1.1880	7.6200	7.6208	5.7979	7.5953	5.2862	7.3795	7.2189
1.1940	7.6730	7.6738	5.9059	7.6493	5.3602	7.4515	7.2949
1.2000	7.7160	7.7208	6.0279	7.7003	5.4342	7.5155	7.3689
1.2060	7.7540	7.7668	6.1699	7.7483	5.5142	7.5805	7.4399
1.2120	7.8060	7.8088	6.3289	7.7923	5.6042	7.6395	7.5079
1.2180	7.8490	7.8568	6.5049	7.8353	5.7052	7.6975	7.5699
1.2240	7.8870	7.8988	6.6849	7.8783	5.8232	7.7525	7.6299
1.2300	7.9220	7.9378	6.8549	7.9193	5.9602	7.8075	7.6869
1.2360	7.9680	7.9768	7.0059	7.9573	6.1192	7.8575	7.7409
1.2420	8.0070	8.0158	7.1349	7.9963	6.3012	7.9055	7.7959

1.2480	8.0480	8.0518	7.2499	8.0343	6.4922	7.9515	7.8439
1.2540	8.0860	8.0888	7.3489	8.0713	6.6752	7.9935	7.8909
1.2600	8.1230	8.1248	7.4359	8.1053	6.8322	8.0355	7.9369
1.2660	8.1600	8.1588	7.5119	8.1413	6.9662	8.0775	7.9809
1.2720	8.1950	8.1918	7.5819	8.1763	7.0692	8.1185	8.0209
1.2780	8.2300	8.2238	7.6439	8.2113	7.1672	8.1575	8.0629
1.2840	8.2650	8.2608	7.7049	8.2443	7.2502	8.1955	8.0619
1.2900	8.3000	8.2928	7.7599	8.2763	7.3272	8.2315	8.1409
1.2960	8.3330	8.3228	7.8109	8.3093	7.3932	8.2695	8.1769
1.3020	8.3670	8.3568	7.8589	8.3423	7.4532	8.3065	8.2159
1.3080	8.3980	8.3878	7.9049	8.3743	7.5122	8.3415	8.2509
1.3140	8.4310	8.4208	7.9479	8.4053	7.5672	8.3745	8.2869
1.3200	8.4640	8.4528	7.9889	8.4373	7.6182	8.4075	8.3209
1.3260	8.4920	8.4828	8.0289	8.4683	7.6632	8.4395	8.3559
1.3320	8.5260	8.5138	8.0679	8.4993	7.7122	8.4725	8.3889
1.3380	8.5550	8.5428	8.1029	8.5293	7.7592	8.5045	8.4219
1.3440	8.5870	8.5728	8.1379	8.5613	7.8052	8.5355	8.4549
1.3500	8.6180	8.6038	8.1739	8.5913	7.8492	8.5655	8.4849
1.3560	8.6480	8.6318	8.2089	8.6203	7.8912	8.5975	8.5159
1.3620	8.6780	8.6598	8.2409	8.6503	7.9312	8.6295	8.5479
1.3680	8.7100	8.6868	8.2729	8.6803	7.9722	8.6585	8.5789
1.3740	8.7390	8.7198	8.3039	8.7113	8.0052	8.6895	8.6089
1.3800	8.7680	8.7478	8.3349	8.7403	8.0472	8.7195	8.6399
1.3860	8.7960	8.7758	8.3649	8.7693	8.0842	8.7495	8.6699
1.3920	8.8260	8.8048	8.3949	8.7983	8.1232	8.7765	8.6999
1.3980	8.8530	8.8348	8.4249	8.8273	8.1572	8.8065	8.7289
1.4040	8.8840	8.8618	8.4549	8.8573	8.1942	8.8355	8.7569
1.4100	8.9140	8.8908	8.4849	8.8863	8.2292	8.8645	8.7869
1.4160	8.9400	8.9198	8.5129	8.9143	8.2622	8.8925	8.8159
1.4220	8.9720	8.9458	8.5419	8.9423	8.2972	8.9225	8.8459
1.4280	9.0000	8.9758	8.5709	8.9703	8.3312	8.9495	8.8739
1.4340	9.0280	9.0048	8.5999	9.0003	8.3642	8.9785	8.9029
1.4400	9.0560	9.0298	8.6299	9.0283	8.3962	9.0055	8.9299
1.4460	9.0860	9.0608	8.6559	9.0563	8.4272	9.0335	8.9579
1.4520	9.1130	9.0898	8.6829	9.0843	8.4602	9.0615	8.9859
1.4580	9.1420	9.1168	8.7119	9.1123	8.4902	9.0895	9.0119
1.4640	9.1690	9.1438	8.7399	9.1403	8.5222	9.1175	9.0399
1.4700	9.1970	9.1698	8.7679	9.1683	8.5522	9.1455	9.0679
1.4760	9.2230	9.1978	8.7949	9.1963	8.5802	9.1725	9.0939
1.4820	9.2520	9.2248	8.8229	9.2243	8.6112	9.2005	9.1219
1.4880	9.2800	9.2548	8.8499	9.2513	8.6432	9.2265	9.1499
1.4940	9.3100	9.2828	8.8769	9.2793	8.6712	9.2525	9.1499
1.5000	9.3320	9.3098	8.9039	9.3063	8.7012	9.2805	9.2039
1.5060	9.3660	9.3378	8.9309	9.3343	8.7302	9.3065	9.2319
1.5120	9.3940	9.3638	8.9589	9.3623	8.7592	9.3325	9.2579
1.5180	9.4220	9.3938	8.9859	9.3913	8.7872	9.3615	9.2849
1.5240	9.4480	9.4208	9.0129	9.4193	8.8162	9.3875	9.3119
1.5300	9.4770	9.4488	9.0389	9.4453	8.8442	9.4135	9.3379
1.5360	9.5060	9.4768	9.0659	9.4713	8.8722	9.4395	9.3639
1.5420	9.5310	9.5028	9.0929	9.4993	8.9012	9.4655	9.3899
1.5480	9.5590	9.5328	9.1199	9.5253	8.9292	9.4935	9.4169

1.5540	9.5840	9.5638	9.1469	9.5553	8.9552	9.5205	9.4429
1.5600	9.6080	9.5858	9.1739	9.5813	8.9832	9.5455	9.4709
1.5660	9.6380	9.6168	9.2009	9.6073	9.0102	9.5695	9.4949
1.5720	9.6650	9.6418	9.2269	9.6333	9.0372	9.5955	9.5199
1.5780	9.6930	9.6698	9.2549	9.6593	9.0632	9.6205	9.5469
1.5840	9.7160	9.6958	9.2799	9.6853	9.0912	9.6445	9.5699
1.5900	9.7440	9.7238	9.3079	9.7113	9.1182	9.6705	9.5959
1.5960	9.7710	9.7508	9.3349	9.7353	9.1412	9.6935	9.6209
1.6020	9.7950	9.7748	9.3609	9.7613	9.1712	9.7175	9.6449
1.6080	9.8210	9.8018	9.3869	9.7863	9.1962	9.7425	9.6699
1.6140	9.8460	9.8278	9.4139	9.8113	9.2242	9.7655	9.6939
1.6200	9.8690	9.8528	9.4389	9.8353	9.2472	9.7885	9.7169
1.6260	9.8950	9.8768	9.4639	9.8583	9.2762	9.8105	9.7409
1.6320	9.9160	9.9018	9.4919	9.8813	9.2992	9.8345	9.7639
1.6380	9.9430	9.9268	9.5169	9.9053	9.3262	9.8555	9.7859
1.6440	9.9650	9.9498	9.5429	9.9283	9.3522	9.8765	9.8089
1.6500	9.9890	9.9748	9.5679	9.9513	9.3752	9.8975	9.8309
1.6560	10.0110	9.9978	9.5939	9.9733	9.4002	9.9215	9.8519
1.6620	10.0320	10.0198	9.6219	9.9963	9.4282	9.9435	9.8739
1.6680	10.0570	10.0438	9.6469	10.0173	9.4532	9.9645	9.8959
1.6740	10.0780	10.0678	9.6709	10.0403	9.4762	9.9855	9.9169
1.6800	10.1010	10.0878	9.6959	10.0613	9.5012	10.0055	9.9379
1.6860	10.1220	10.1098	9.7219	10.0823	9.5212	10.0275	9.9599
1.6920	10.1430	10.1328	9.7449	10.1033	9.5492	10.0465	9.9809
1.6980	10.1620	10.1548	9.7719	10.1243	9.5762	10.0655	10.0009
1.7040	10.1840	10.1748	9.7969	10.1453	9.5992	10.0845	10.0219
1.7100	10.2040	10.1988	9.8199	10.1663	9.6222	10.1055	10.0409
1.7160	10.2240	10.2198	9.8449	10.1853	9.6452	10.1255	10.0609
1.7220	10.2410	10.2408	9.8679	10.2063	9.6682	10.1445	10.0819
1.7280	10.2620	10.2618	9.8909	10.2253	9.6932	10.1635	10.1009
1.7340	10.2830	10.2828	9.9149	10.2453	9.7142	10.1805	10.1199
1.7400	10.3030	10.3028	9.9389	10.2643	9.7372	10.2005	10.1389
1.7460	10.3210	10.3218	9.9619	10.2833	9.7612	10.2185	10.1579
1.7520	10.3420	10.3418	9.9849	10.3033	9.7832	10.2365	10.1769
1.7580	10.3610	10.3608	10.0079	10.3223	9.8042	10.2545	10.1969
1.7640	10.3800	10.3808	10.0299	10.3413	9.8282	10.2735	10.2139
1.7700	10.3970	10.4018	10.0539	10.3603	9.8512	10.2905	10.2329
1.7760	10.4170	10.4208	10.0749	10.3783	9.8722	10.3085	10.2509
1.7820	10.4340	10.4398	10.0969	10.3993	9.8942	10.3275	10.2699
1.7880	10.4520	10.4578	10.1189	10.4173	9.9152	10.3455	10.2869
1.7940	10.4720	10.4768	10.1399	10.4353	9.9362	10.3615	10.3059
1.8000	10.4880	10.4948	10.1609	10.4533	9.9582	10.3805	10.3249
1.8060	10.5050	10.5128	10.1829	10.4703	9.9782	10.3965	10.3409
1.8120	10.5230	10.5298	10.2019	10.4863	10.0002	10.4115	10.3589
1.8180	10.5410	10.5458	10.2249	10.5033	10.0192	10.4295	10.3749
1.8240	10.5580	10.5638	10.2449	10.5203	10.0402	10.4455	10.3919
1.8300	10.5730	10.5808	10.2659	10.5373	10.0562	10.4605	10.4099
1.8360	10.5890	10.5988	10.2859	10.5533	10.0792	10.4765	10.4249
1.8420	10.6050	10.6148	10.3049	10.5693	10.0992	10.4915	10.4389
1.8480	10.6210	10.6318	10.3239	10.5843	10.1142	10.5065	10.4569
1.8540	10.6360	10.6488	10.3439	10.6013	10.1362	10.5225	10.4719

1.8600	10.6530	10.6648	10.3629	10.6163	10.1572	10.5375	10.4879
1.8660	10.6680	10.6788	10.3809	10.6323	10.1712	10.5515	10.5019
1.8720	10.6830	10.6948	10.4009	10.6473	10.1922	10.5675	10.5159
1.8780	10.6970	10.7108	10.4199	10.6633	10.2102	10.5835	10.5329
1.8840	10.7110	10.7258	10.4399	10.6773	10.2262	10.5975	10.5489
1.8900	10.7250	10.7398	10.4589	10.6923	10.2502	10.6115	10.5629
1.8960	10.7410	10.7558	10.4769	10.7073	10.2652	10.6275	10.5789
1.9020	10.7550	10.7688	10.4939	10.7213	10.2822	10.6415	10.5929
1.9080	10.7670	10.7838	10.5119	10.7353	10.3022	10.6555	10.6079
1.9140	10.7810	10.7968	10.5299	10.7503	10.3172	10.6695	10.6219
1.9200	10.7950	10.8108	10.5459	10.7633	10.3292	10.6825	10.6359
1.9260	10.8090	10.8248	10.5639	10.7773	10.3512	10.6955	10.6499
1.9320	10.8230	10.8388	10.5799	10.7913	10.3692	10.7095	10.6639
1.9380	10.8370	10.8528	10.5969	10.8033	10.3892	10.7235	10.6779
1.9440	10.8490	10.8668	10.6129	10.8173	10.4062	10.7365	10.6899
1.9500	10.8620	10.8798	10.6289	10.8293	10.4202	10.7495	10.7059
1.9560	10.8720	10.8918	10.6449	10.8423	10.4362	10.7615	10.7189
1.9620	10.8870	10.9058	10.6619	10.8553	10.4542	10.7755	10.7329
1.9680	10.8990	10.9188	10.6779	10.8683	10.4692	10.7875	10.7459
1.9740	10.9100	10.9308	10.6939	10.8803	10.4842	10.8005	10.7589
1.9800	10.9220	10.9428	10.7089	10.8933	10.5012	10.8105	10.7709
1.9860	10.9340	10.9558	10.7249	10.9053	10.5162	10.8245	10.7849
1.9920	10.9480	10.9678	10.7399	10.9173	10.5322	10.8385	10.7969
1.9980		10.9798	10.7559	10.9293	10.5462	10.8495	10.8089
2.0040			10.7709	10.9413	10.5622	10.8625	10.8219
2.0100			10.7849	10.9523	10.5762	10.8735	10.8329
2.0160			10.8009	10.9643	10.5922	10.8855	10.8449
2.0220			10.8149		10.6062	10.8975	10.8559
2.0280			10.8289		10.6182	10.9085	10.8689
2.0340			10.8429		10.6352	10.9195	10.8799
2.0400			10.8569		10.6502	10.9295	10.8909
2.0460			10.8699		10.6642	10.9415	10.9029
2.0520			10.8839		10.6772	10.9525	10.9159
2.0580			10.8979		10.6882	10.9625	10.9259
2.0640			10.9099		10.7062		10.9359
2.0700			10.9239		10.7202		10.9479
2.0760			10.9369		10.7322		
2.0820			10.9489		10.7462		
2.0880			10.9619		10.7592		
2.0940			10.9739		10.7732		
2.1000			10.9859		10.7852		
2.1060			10.9989		10.7972		
2.1120					10.8092		
2.1180					10.8222		
2.1240					10.8342		
2.1300					10.8472		
2.1360					10.8592		
2.1420					10.8712		
2.1480					10.8812		
2.1540					10.8932		
2.1600					10.9042		

2.1660					10.9152		
2.1720					10.9272		
2.1780					10.9382		
2.1840					10.9492		
2.1900					10.9602		

Table S5. Data obtained from potentiometric measurements during studies of lanthanide ion/glycolic acid/spermine ternary systems.

V [ml]	pH						
	La(III)	Nd(III)	Eu(III)	Gd(III)	Tb(III)	Ho(III)	Lu(III)
0.0000	2.3604	2.3684	2.3484	2.3694	2.3011	2.3694	2.3244
0.0060	2.3644	2.3714	2.3524	2.3724	2.3031	2.3724	2.3284
0.0120	2.3674	2.3744	2.3574	2.3754	2.3061	2.3754	2.3324
0.0180	2.3714	2.3784	2.3614	2.3794	2.3111	2.3794	2.3364
0.0240	2.3754	2.3824	2.3664	2.3834	2.3161	2.3824	2.3404
0.0300	2.3784	2.3854	2.3714	2.3864	2.3201	2.3854	2.3444
0.0360	2.3824	2.3894	2.3764	2.3914	2.3241	2.3894	2.3484
0.0420	2.3874	2.3924	2.3804	2.3954	2.3291	2.3924	2.3524
0.0480	2.3914	2.3964	2.3854	2.3994	2.3331	2.3964	2.3564
0.0540	2.3964	2.4014	2.3894	2.4034	2.3371	2.4004	2.3604
0.0600	2.3994	2.4044	2.3944	2.4074	2.3421	2.4054	2.3644
0.0660	2.4034	2.4084	2.3984	2.4114	2.3471	2.4084	2.3684
0.0720	2.4084	2.4134	2.4034	2.4154	2.3511	2.4114	2.3734
0.0780	2.4124	2.4174	2.4084	2.4204	2.3561	2.4164	2.3764
0.0840	2.4164	2.4214	2.4134	2.4244	2.3601	2.4204	2.3804
0.0900	2.4204	2.4254	2.4174	2.4304	2.3651	2.4244	2.3854
0.0960	2.4274	2.4314	2.4224	2.4344	2.3691	2.4304	2.3894
0.1020	2.4314	2.4344	2.4294	2.4394	2.3741	2.4334	2.3924
0.1080	2.4364	2.4394	2.4344	2.4434	2.3791	2.4384	2.3974
0.1140	2.4414	2.4434	2.4394	2.4484	2.3841	2.4424	2.4004
0.1200	2.4464	2.4484	2.4434	2.4524	2.3881	2.4464	2.4064
0.1260	2.4504	2.4534	2.4494	2.4574	2.3931	2.4504	2.4104
0.1320	2.4554	2.4574	2.4534	2.4624	2.3981	2.4554	2.4154
0.1380	2.4604	2.4624	2.4594	2.4674	2.4021	2.4604	2.4204
0.1440	2.4654	2.4684	2.4644	2.4724	2.4081	2.4644	2.4244
0.1500	2.4714	2.4734	2.4694	2.4764	2.4151	2.4694	2.4314
0.1560	2.4764	2.4784	2.4754	2.4824	2.4201	2.4744	2.4364
0.1620	2.4814	2.4824	2.4804	2.4874	2.4251	2.4784	2.4404
0.1680	2.4874	2.4874	2.4854	2.4924	2.4311	2.4824	2.4454
0.1740	2.4924	2.4924	2.4904	2.4974	2.4361	2.4874	2.4504
0.1800	2.4974	2.4974	2.4964	2.5024	2.4411	2.4914	2.4544
0.1860	2.5014	2.5024	2.5014	2.5064	2.4451	2.4964	2.4594
0.1920	2.5074	2.5084	2.5064	2.5124	2.4501	2.5014	2.4644
0.1980	2.5124	2.5124	2.5114	2.5174	2.4561	2.5064	2.4684
0.2040	2.5174	2.5184	2.5174	2.5224	2.4611	2.5104	2.4734
0.2100	2.5234	2.5244	2.5234	2.5274	2.4671	2.5154	2.4784
0.2160	2.5284	2.5294	2.5294	2.5334	2.4721	2.5204	2.4834
0.2220	2.5344	2.5344	2.5344	2.5394	2.4771	2.5254	2.4894

0.2280	2.5394	2.5394	2.5394	2.5444	2.4831	2.5314	2.4944
0.2340	2.5454	2.5454	2.5454	2.5494	2.4891	2.5364	2.4994
0.2400	2.5514	2.5504	2.5524	2.5574	2.4951	2.5414	2.5044
0.2460	2.5574	2.5564	2.5574	2.5614	2.5001	2.5464	2.5094
0.2520	2.5624	2.5614	2.5644	2.5684	2.5051	2.5524	2.5154
0.2580	2.5694	2.5684	2.5694	2.5734	2.5121	2.5574	2.5184
0.2640	2.5754	2.5734	2.5764	2.5794	2.5181	2.5624	2.5254
0.2700	2.5814	2.5794	2.5824	2.5854	2.5241	2.5684	2.5314
0.2760	2.5874	2.5854	2.5894	2.5924	2.5301	2.5744	2.5364
0.2820	2.5944	2.5914	2.5954	2.5994	2.5361	2.5804	2.5414
0.2880	2.6004	2.5984	2.6014	2.6044	2.5431	2.5854	2.5474
0.2940	2.6064	2.6044	2.6084	2.6114	2.5501	2.5924	2.5534
0.3000	2.6134	2.6114	2.6154	2.6174	2.5561	2.5974	2.5594
0.3060	2.6204	2.6174	2.6224	2.6244	2.5621	2.6044	2.5644
0.3120	2.6274	2.6244	2.6294	2.6314	2.5691	2.6094	2.5714
0.3180	2.6344	2.6314	2.6364	2.6384	2.5761	2.6154	2.5764
0.3240	2.6414	2.6384	2.6434	2.6454	2.5821	2.6214	2.5834
0.3300	2.6484	2.6454	2.6504	2.6514	2.5891	2.6284	2.5884
0.3360	2.6554	2.6524	2.6574	2.6594	2.5961	2.6344	2.5964
0.3420	2.6624	2.6594	2.6644	2.6664	2.6031	2.6404	2.6014
0.3480	2.6694	2.6664	2.6714	2.6734	2.6101	2.6474	2.6084
0.3540	2.6774	2.6734	2.6784	2.6814	2.6181	2.6534	2.6144
0.3600	2.6844	2.6804	2.6864	2.6884	2.6251	2.6604	2.6204
0.3660	2.6924	2.6874	2.6944	2.6954	2.6321	2.6674	2.6274
0.3720	2.6994	2.6964	2.7024	2.7034	2.6401	2.6744	2.6344
0.3780	2.7084	2.7034	2.7104	2.7114	2.6471	2.6814	2.6404
0.3840	2.7154	2.7104	2.7174	2.7184	2.6561	2.6884	2.6474
0.3900	2.7234	2.7184	2.7254	2.7274	2.6631	2.6964	2.6554
0.3960	2.7324	2.7264	2.7344	2.7354	2.6711	2.7034	2.6624
0.4020	2.7394	2.7344	2.7434	2.7434	2.6791	2.7104	2.6694
0.4080	2.7494	2.7434	2.7524	2.7524	2.6881	2.7184	2.6764
0.4140	2.7584	2.7514	2.7604	2.7614	2.6951	2.7254	2.6834
0.4200	2.7664	2.7604	2.7694	2.7704	2.7061	2.7344	2.6904
0.4260	2.7754	2.7694	2.7784	2.7794	2.7141	2.7414	2.6974
0.4320	2.7854	2.7784	2.7884	2.7884	2.7231	2.7494	2.7064
0.4380	2.7954	2.7874	2.7974	2.7974	2.7321	2.7574	2.7134
0.4440	2.8054	2.7974	2.8074	2.8084	2.7411	2.7664	2.7224
0.4500	2.8144	2.8064	2.8174	2.8174	2.7511	2.7754	2.7294
0.4560	2.8244	2.8174	2.8274	2.8274	2.7611	2.7834	2.7384
0.4620	2.8344	2.8264	2.8384	2.8384	2.7701	2.7924	2.7464
0.4680	2.8454	2.8364	2.8484	2.8484	2.7801	2.8014	2.7544
0.4740	2.8554	2.8464	2.8594	2.8594	2.7911	2.8104	2.7644
0.4800	2.8674	2.8574	2.8694	2.8704	2.8021	2.8204	2.7724
0.4860	2.8784	2.8674	2.8814	2.8824	2.8131	2.8294	2.7824
0.4920	2.8904	2.8794	2.8924	2.8934	2.8241	2.8394	2.7904
0.4980	2.9024	2.8904	2.9044	2.9064	2.8351	2.8504	2.8004
0.5040	2.9134	2.9014	2.9164	2.9174	2.8471	2.8604	2.8104
0.5100	2.9264	2.9134	2.9284	2.9294	2.8581	2.8704	2.8184
0.5160	2.9394	2.9264	2.9414	2.9414	2.8711	2.8804	2.8294
0.5220	2.9524	2.9384	2.9534	2.9534	2.8831	2.8914	2.8394
0.5280	2.9644	2.9524	2.9664	2.9674	2.8961	2.9024	2.8494

0.5340	2.9784	2.9644	2.9804	2.9814	2.9081	2.9134	2.8594
0.5400	2.9934	2.9774	2.9944	2.9954	2.9201	2.9254	2.8694
0.5460	3.0064	2.9904	3.0084	3.0094	2.9361	2.9364	2.8814
0.5520	3.0204	3.0044	3.0234	3.0244	2.9501	2.9484	2.8924
0.5580	3.0364	3.0184	3.0374	3.0404	2.9641	2.9604	2.9044
0.5640	3.0524	3.0324	3.0544	3.0554	2.9791	2.9734	2.9154
0.5700	3.0684	3.0504	3.0694	3.0714	2.9891	2.9874	2.9274
0.5760	3.0854	3.0644	3.0854	3.0894	2.9891	2.9994	2.9394
0.5820	3.1014	3.0804	3.1014	3.1044	2.9891	3.0134	2.9524
0.5880	3.1184	3.0964	3.1184	3.1224	2.9891	3.0264	2.9654
0.5940	3.1354	3.1144	3.1364	3.1404	2.9901	3.0404	2.9784
0.6000	3.1544	3.1314	3.1534	3.1584	2.9891	3.0554	2.9904
0.6060	3.1724	3.1484	3.1734	3.1784	2.9951	3.0704	3.0044
0.6120	3.1924	3.1654	3.1924	3.1974	3.0121	3.0854	3.0184
0.6180	3.2114	3.1834	3.2114	3.2174	3.0271	3.0994	3.0324
0.6240	3.2314	3.2034	3.2324	3.2394	3.0441	3.1154	3.0464
0.6300	3.2524	3.2204	3.2524	3.2604	3.0611	3.1324	3.0624
0.6360	3.2764	3.2414	3.2744	3.2854	3.0781	3.1484	3.0774
0.6420	3.2984	3.2624	3.2974	3.3074	3.0961	3.1664	3.0924
0.6480	3.3224	3.2864	3.3204	3.3314	3.1141	3.1834	3.1084
0.6540	3.3444	3.3094	3.3434	3.3554	3.1321	3.2014	3.1254
0.6600	3.3694	3.3314	3.3684	3.3814	3.1521	3.2204	3.1414
0.6660	3.3944	3.3534	3.3934	3.4094	3.1731	3.2384	3.1584
0.6720	3.4194	3.3764	3.4184	3.4364	3.1941	3.2584	3.1764
0.6780	3.4464	3.4004	3.4464	3.4644	3.2151	3.2804	3.1954
0.6840	3.4734	3.4274	3.4744	3.4964	3.2371	3.3004	3.2144
0.6900	3.5034	3.4534	3.5024	3.5244	3.2601	3.3224	3.2334
0.6960	3.5304	3.4804	3.5314	3.5574	3.2861	3.3434	3.2524
0.7020	3.5624	3.5074	3.5624	3.5924	3.3101	3.3654	3.2724
0.7080	3.5934	3.5364	3.5944	3.6254	3.3361	3.3884	3.2944
0.7140	3.6254	3.5664	3.6274	3.6624	3.3621	3.4114	3.3164
0.7200	3.6604	3.5974	3.6624	3.7014	3.3901	3.4364	3.3384
0.7260	3.6954	3.6294	3.6984	3.7424	3.4191	3.4614	3.3604
0.7320	3.7324	3.6624	3.7364	3.7854	3.4491	3.4874	3.3844
0.7380	3.7714	3.6974	3.7774	3.8314	3.4791	3.5144	3.4094
0.7440	3.8114	3.7344	3.8204	3.8814	3.5131	3.5434	3.4364
0.7500	3.8554	3.7744	3.8644	3.9364	3.5481	3.5724	3.4624
0.7560	3.9014	3.8144	3.9134	3.9944	3.5851	3.6024	3.4884
0.7620	3.9484	3.8574	3.9664	4.0594	3.6231	3.6344	3.5184
0.7680	4.0014	3.9044	4.0234	4.1304	3.6641	3.6664	3.5484
0.7740	4.0574	3.9534	4.0854	4.2124	3.7081	3.7004	3.5794
0.7800	4.1184	4.0044	4.1554	4.3064	3.7531	3.7364	3.6114
0.7860	4.1874	4.0624	4.2314	4.4164	3.8011	3.7734	3.6444
0.7920	4.2604	4.1274	4.3184	4.5554	3.8551	3.8124	3.6804
0.7980	4.3464	4.1974	4.4224	4.7444	3.9131	3.8544	3.7174
0.8040	4.4444	4.2744	4.5504	5.0504	3.9761	3.8974	3.7554
0.8100	4.5664	4.3664	4.7204	5.6394	4.0451	3.9444	3.7954
0.8160	4.7184	4.4714	4.9764	6.3584	4.1251	3.9934	3.8394
0.8220	4.9454	4.6024	5.4364	6.7834	4.2171	4.0464	3.8854
0.8280	5.3354	4.7744	6.2204	7.0074	4.3271	4.1024	3.9324
0.8340	6.0574	5.0524	6.7244	7.1534	4.4591	4.1694	3.9854

0.8400	6.7094	5.5454	6.9814	7.2564	4.6361	4.2374	4.0414
0.8460	7.0414	6.2854	7.1374	7.3374	4.9131	4.3174	4.1024
0.8520	7.2414	6.7524	7.2534	7.4044	5.4471	4.4084	4.1734
0.8580	7.3874	7.0074	7.3394	7.4634	6.1951	4.5184	4.2504
0.8640	7.4984	7.1844	7.4124	7.5104	6.5471	4.6574	4.3384
0.8700	7.5914	7.3044	7.4744	7.5584	6.7051	4.8484	4.4424
0.8760	7.6674	7.4004	7.5234	7.5984	6.8461	5.1514	4.5724
0.8820	7.7344	7.4774	7.5714	7.6324	6.9731	5.6974	4.7484
0.8880	7.7954	7.5494	7.6094	7.6634	7.0771	6.3814	5.0064
0.8940	7.8554	7.6084	7.6444	7.6904	7.1621	6.7954	5.4854
0.9000	7.9074	7.6584	7.6754	7.7184	7.2401	7.0024	6.1534
0.9060	7.9584	7.7074	7.7004	7.7424	7.3011	7.1324	6.6274
0.9120	8.0024	7.7514	7.7264	7.7614	7.3521	7.2234	6.8494
0.9180	8.0494	7.7914	7.7464	7.7794	7.3951	7.2914	6.9674
0.9240	8.0904	7.8264	7.7634	7.7974	7.4281	7.3424	7.0444
0.9300	8.1304	7.8584	7.7824	7.8124	7.4581	7.3824	7.0974
0.9360	8.1684	7.8864	7.7794	7.8254	7.4881	7.4104	7.1404
0.9420	8.2014	7.9144	7.7934	7.8404	7.5121	7.4424	7.1764
0.9480	8.2374	7.9394	7.8074	7.8534	7.5311	7.4674	7.2024
0.9540	8.2684	7.9584	7.8054	7.8644	7.5521	7.4894	7.2284
0.9600	8.3004	7.9784	7.8214	7.8754	7.5681	7.5094	7.2514
0.9660	8.3274	7.9944	7.8374	7.8504	7.5831	7.5334	7.2714
0.9720	8.3534	8.0094	7.8294	7.8654	7.5951	7.5444	7.2884
0.9780	8.3814	8.0264	7.8424	7.8814	7.5951	7.5614	7.3084
0.9840	8.4044	8.0394	7.8584	7.8964	7.6201	7.5764	7.3234
0.9900	8.4274	8.0504	7.8664	7.9094	7.6291	7.5914	7.3404
0.9960	8.4494	8.0624	7.8794	7.9004	7.6311	7.6074	7.3564
1.0020	8.4674	8.0754	7.8954	7.9144	7.6371	7.6194	7.3724
1.0080	8.4864	8.0844	7.9134	7.9284	7.6441	7.6324	7.3844
1.0140	8.5064	8.0974	7.9234	7.9404	7.6351	7.6444	7.3984
1.0200	8.5214	8.1074	7.9104	7.9534	7.6461	7.6544	7.4134
1.0260	8.5394	8.1164	7.9284	7.9674	7.6571	7.6694	7.4254
1.0320	8.5564	8.1294	7.9434	7.9834	7.6701	7.6834	7.4404
1.0380	8.5734	8.1384	7.9654	7.9814	7.6811	7.6934	7.4534
1.0440	8.5884	8.1514	7.9814	7.9954	7.6801	7.7024	7.4654
1.0500	8.6024	8.1454	7.9974	8.0134	7.7001	7.6964	7.4794
1.0560	8.6174	8.1584	8.0134	8.0274	7.6871	7.7114	7.4934
1.0620	8.6324	8.1744	8.0104	8.0354	7.6961	7.7144	7.5054
1.0680	8.6464	8.1894	8.0264	8.0494	7.7091	7.7274	7.5214
1.0740	8.6594	8.2024	8.0444	8.0644	7.7181	7.7424	7.5344
1.0800	8.6724	8.2024	8.0634	8.0814	7.7271	7.7584	7.5374
1.0860	8.6884	8.2334	8.0814	8.0974	7.7371	7.7664	7.5534
1.0920	8.7034	8.2474	8.0954	8.1114	7.7461	7.7804	7.5654
1.0980	8.7174	8.2594	8.1084	8.1214	7.7581	7.7934	7.5764
1.1040	8.7334	8.2724	8.1254	8.1354	7.7581	7.8094	7.5934
1.1100	8.7494	8.2874	8.1354	8.1514	7.7681	7.8244	7.6034
1.1160	8.7654	8.3024	8.1514	8.1684	7.7781	7.8374	7.6234
1.1220	8.7814	8.3174	8.1674	8.1854	7.7821	7.8534	7.6434
1.1280	8.8004	8.3334	8.1864	8.2034	7.7931	7.8524	7.6634
1.1340	8.8164	8.3474	8.2074	8.2214	7.8061	7.8614	7.6864
1.1400	8.8334	8.3634	8.2264	8.2394	7.8191	7.8834	7.7134

1.1460	8.8514	8.3814	8.2474	8.2564	7.8341	7.9034	7.7184
1.1520	8.8724	8.3954	8.2654	8.2754	7.8451	7.9254	7.7464
1.1580	8.8914	8.4114	8.2864	8.2964	7.8531	7.9424	7.7744
1.1640	8.9094	8.4274	8.3084	8.3164	7.8621	7.9604	7.8064
1.1700	8.9284	8.4434	8.3304	8.3344	7.8731	7.9834	7.8354
1.1760	8.9474	8.4584	8.3534	8.3564	7.8831	8.0054	7.8634
1.1820	8.9674	8.4744	8.3774	8.3794	7.8941	8.0324	7.8974
1.1880	8.9844	8.4924	8.4024	8.4004	7.8951	8.0564	7.9264
1.1940	9.0044	8.5084	8.4254	8.4214	7.9251	8.0804	7.9604
1.2000	9.0234	8.5264	8.4494	8.4474	7.9371	8.1054	7.9974
1.2060	9.0404	8.5424	8.4774	8.4694	7.9521	8.1324	8.0354
1.2120	9.0614	8.5614	8.5034	8.4954	7.9661	8.1604	8.0684
1.2180	9.0814	8.5794	8.5284	8.5204	7.9801	8.1864	8.1054
1.2240	9.1004	8.5984	8.5564	8.5484	7.9931	8.2164	8.1444
1.2300	9.1194	8.6164	8.5844	8.5744	8.0061	8.2494	8.1824
1.2360	9.1374	8.6354	8.6124	8.6034	8.0241	8.2814	8.2254
1.2420	9.1574	8.6534	8.6424	8.6324	8.0391	8.3164	8.2614
1.2480	9.1754	8.6724	8.6734	8.6604	8.0601	8.3514	8.3004
1.2540	9.1954	8.6914	8.7034	8.6904	8.0751	8.3884	8.3404
1.2600	9.2124	8.7114	8.7354	8.7194	8.0941	8.4264	8.3794
1.2660	9.2314	8.7304	8.7664	8.7494	8.1111	8.4614	8.4164
1.2720	9.2474	8.7534	8.7984	8.7814	8.1321	8.5004	8.4514
1.2780	9.2684	8.7744	8.8324	8.8134	8.1501	8.5384	8.4914
1.2840	9.2864	8.7944	8.8644	8.8444	8.1721	8.5744	8.5234
1.2900	9.3034	8.8164	8.8974	8.8764	8.1911	8.6134	8.5594
1.2960	9.3194	8.8394	8.9324	8.9094	8.2121	8.6504	8.5964
1.3020	9.3354	8.8614	8.9654	8.9404	8.2321	8.6904	8.6324
1.3080	9.3534	8.8634	8.9994	8.9744	8.2571	8.7284	8.6674
1.3140	9.3714	8.9124	9.0334	9.0054	8.2821	8.7654	8.6994
1.3200	9.3874	8.9394	9.0684	9.0374	8.3081	8.8034	8.7354
1.3260	9.4054	8.9654	9.1044	9.0724	8.3331	8.8384	8.7704
1.3320	9.4234	8.9924	9.1384	9.1044	8.3611	8.8774	8.8034
1.3380	9.4384	9.0194	9.1734	9.1384	8.3861	8.9164	8.8374
1.3440	9.4574	9.0494	9.2094	9.1734	8.4151	8.9544	8.8704
1.3500	9.4754	9.0794	9.2444	9.2074	8.4451	8.9914	8.9054
1.3560	9.4954	9.1084	9.2824	9.2424	8.4751	9.0284	8.9384
1.3620	9.5144	9.1384	9.3194	9.2774	8.5031	9.0654	8.9704
1.3680	9.5304	9.1714	9.3544	9.3124	8.5361	9.1044	9.0034
1.3740	9.5524	9.2034	9.3914	9.3474	8.5661	9.1424	9.0404
1.3800	9.5724	9.2374	9.4284	9.3834	8.5971	9.1814	9.0724
1.3860	9.5934	9.2714	9.4654	9.4174	8.6281	9.2204	9.1074
1.3920	9.6144	9.3044	9.5024	9.4544	8.6581	9.2604	9.1404
1.3980	9.6354	9.3394	9.5394	9.4914	8.6921	9.3004	9.1754
1.4040	9.6604	9.3724	9.5774	9.5264	8.7251	9.3394	9.2124
1.4100	9.6814	9.4094	9.6164	9.5614	8.7591	9.3824	9.2494
1.4160	9.7074	9.4444	9.6524	9.6014	8.7891	9.4214	9.2844
1.4220	9.7344	9.4804	9.6894	9.6374	8.8231	9.4604	9.3174
1.4280	9.7604	9.5174	9.7284	9.6744	8.8571	9.5024	9.3544
1.4340	9.7884	9.5544	9.7674	9.7124	8.8911	9.5454	9.3914
1.4400	9.8174	9.5934	9.8034	9.7494	8.9241	9.5864	9.4284
1.4460	9.8474	9.6304	9.8404	9.7864	8.9581	9.6244	9.4654

1.4520	9.8764	9.6654	9.8784	9.8234	8.9921	9.6684	9.5034
1.4580	9.9094	9.7034	9.9144	9.8584	9.0261	9.7104	9.5394
1.4640	9.9384	9.7374	9.9524	9.8934	9.0571	9.7494	9.5794
1.4700	9.9704	9.7764	9.9844	9.9294	9.0931	9.7914	9.6144
1.4760	10.0014	9.8124	10.0194	9.9634	9.1261	9.8294	9.6534
1.4820	10.0334	9.8464	10.0534	9.9964	9.1611	9.8674	9.6894
1.4880	10.0664	9.8824	10.0884	10.0304	9.1971	9.9074	9.7264
1.4940	10.0984	9.9164	10.1194	10.0654	9.2311	9.9454	9.7624
1.5000	10.1264	9.9524	10.1524	10.0984	9.2681	9.9844	9.8014
1.5060	10.1594	9.9864	10.1864	10.1284	9.3061	10.0234	9.8374
1.5120	10.1884	10.0194	10.2144	10.1564	9.3411	10.0584	9.8744
1.5180	10.2214	10.0514	10.2444	10.1894	9.3761	10.0964	9.9084
1.5240	10.2514	10.0864	10.2734	10.2214	9.4121	10.1304	9.9464
1.5300	10.2804	10.1164	10.3024	10.2514	9.4491	10.1634	9.9804
1.5360	10.3054	10.1474	10.3304	10.2784	9.4841	10.1964	10.0144
1.5420	10.3334	10.1774	10.3564	10.3054	9.5221	10.2304	10.0464
1.5480	10.3624	10.2074	10.3834	10.3334	9.5611	10.2634	10.0844
1.5540	10.3904	10.2354	10.4104	10.3594	9.5981	10.2934	10.1144
1.5600	10.4164	10.2654	10.4334	10.3874	9.6341	10.3224	10.1454
1.5660	10.4404	10.2914	10.4634	10.4144	9.6721	10.3504	10.1774
1.5720	10.4654	10.3174	10.4864	10.4384	9.7061	10.3794	10.2074
1.5780	10.4914	10.3454	10.5104	10.4644	9.7431	10.4074	10.2374
1.5840	10.5154	10.3704	10.5344	10.4874	9.7811	10.4334	10.2664
1.5900	10.5384	10.3964	10.5594	10.5124	9.8151	10.4614	10.2954
1.5960	10.5634	10.4224	10.5814	10.5354	9.8541	10.4874	10.3214
1.6020	10.5864	10.4474	10.6014	10.5584	9.8891	10.5124	10.3494
1.6080	10.6074	10.4714	10.6234	10.5804	9.9231	10.5374	10.3754
1.6140	10.6284	10.4934	10.6454	10.6034	9.9591	10.5614	10.4014
1.6200	10.6524	10.5184	10.6664	10.6264	9.9921	10.5864	10.4284
1.6260	10.6714	10.5404	10.6854	10.6474	10.0261	10.6104	10.4544
1.6320	10.6934	10.5624	10.7074	10.6664	10.0611	10.6334	10.4774
1.6380	10.7144	10.5844	10.7254	10.6864	10.0951	10.6554	10.5014
1.6440	10.7334	10.6074	10.7454	10.7054	10.1251	10.6774	10.5254
1.6500	10.7544	10.6264	10.7654	10.7264	10.1591	10.6974	10.5474
1.6560	10.7704	10.6474	10.7844	10.7454	10.1891	10.7174	10.5714
1.6620	10.7904	10.6664	10.8034	10.7634	10.2191	10.7384	10.5934
1.6680	10.8074	10.6874	10.8194	10.7824	10.2491	10.7574	10.6154
1.6740	10.8254	10.7074	10.8364	10.8004	10.2761	10.7774	10.6364
1.6800	10.8444	10.7264	10.8544	10.8194	10.3061	10.7964	10.6564
1.6860	10.8594	10.7434	10.8724	10.8364	10.3311	10.8154	10.6774
1.6920	10.8774	10.7634	10.8894	10.8544	10.3571	10.8334	10.6974
1.6980	10.8934	10.7804	10.9054	10.8704	10.3841	10.8504	10.7164
1.7040	10.9124	10.7984	10.9244	10.8874	10.4091	10.8684	10.7374
1.7100	10.9284	10.8174	10.9414	10.9034	10.4351	10.8844	10.7554
1.7160	10.9424	10.8324	10.9564	10.9214	10.4601	10.9014	10.7734
1.7220	10.9574	10.8504	10.9704	10.9364	10.4871	10.9194	10.7914
1.7280	10.9724	10.8664	10.9854	10.9524	10.5121	10.9364	10.8104
1.7340		10.8804		10.9664	10.5371	10.9524	10.8274
1.7400		10.8984		10.9824	10.5601	10.9684	10.8434
1.7460		10.9164			10.5821	10.9834	10.8614
1.7520		10.9314			10.6051		10.8774

1.7580		10.9454			10.6281		10.8934	
1.7640		10.9614			10.6501		10.9104	
1.7700		10.9774			10.6711		10.9284	
1.7760					10.6931		10.9424	
1.7820					10.7121		10.9564	
1.7880					10.7331		10.9724	
1.7940					10.7541			
1.8000					10.7761			
1.8060					10.7941			
1.8120					10.8131			
1.8180					10.8311			
1.8240					10.8491			
1.8300					10.8661			
1.8360					10.8861			
1.8420					10.9021			
1.8480					10.9191			

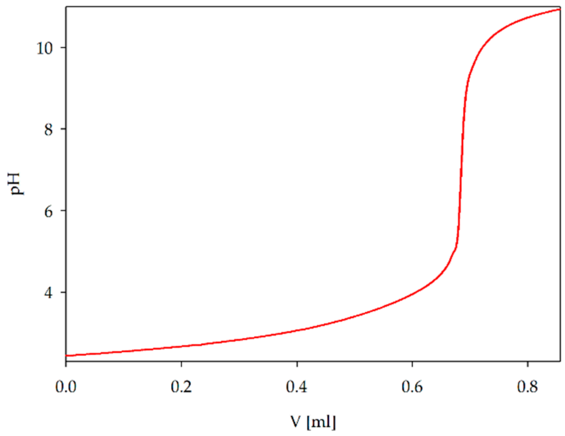

Figure S1. The titration curve for glycolic acid measurements.

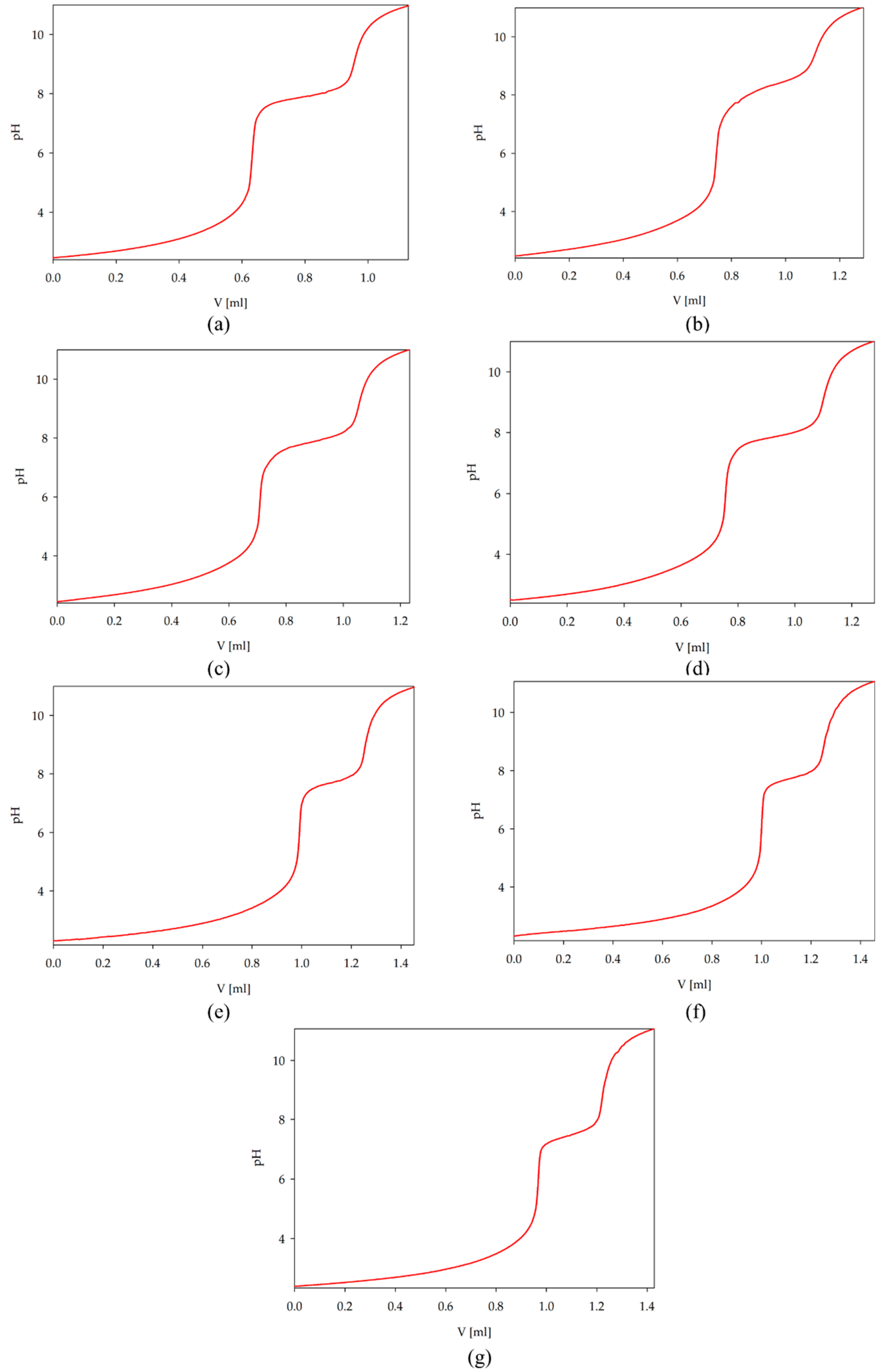

Figure S2. The titration curve for the systems: a) La(III)/GA; b) Nd(III)/GA; c) Eu(III)/GA; d) Gd(III)/GA; e) Tb(III)/GA; f) Ho(III)/GA; g) Lu(III)/GA.

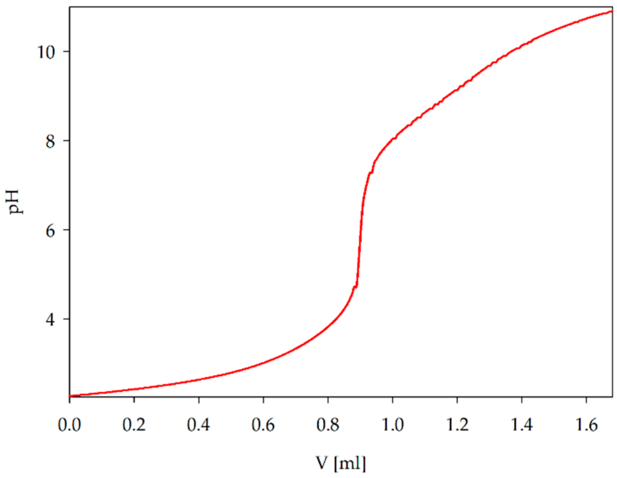

Figure S3. The titration curve for the system GA/Spm.

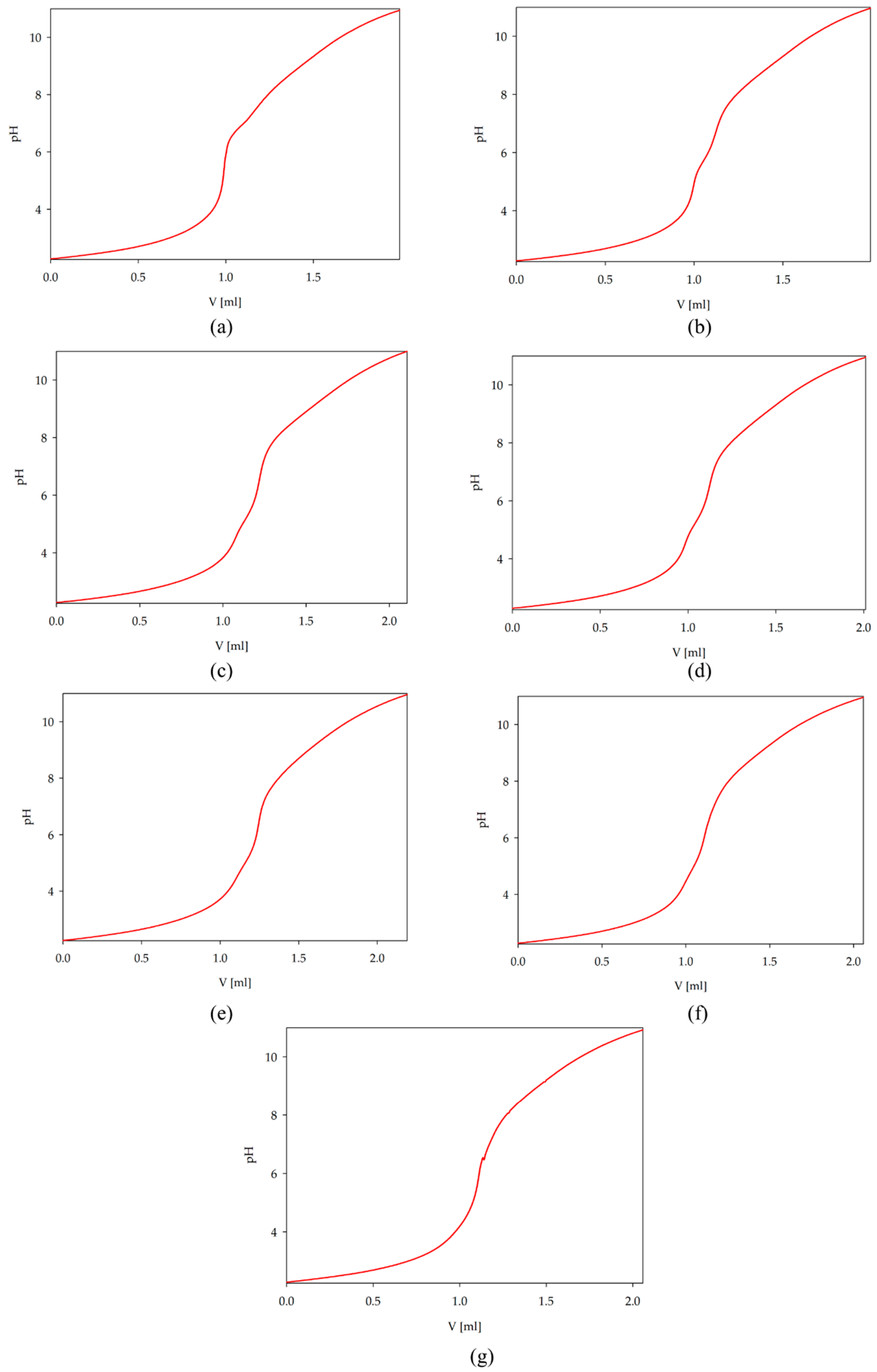

Figure S4. The titration curve for the systems: a) La(III)/Tar/Spm; b) Nd(III)/Tar/Spm; c) Eu(III)/Tar/Spm; d) Tb(III)/Tar/Spm; e) Ho(III)/Tar/Spm; f) Lu(III)/Tar/Spm.

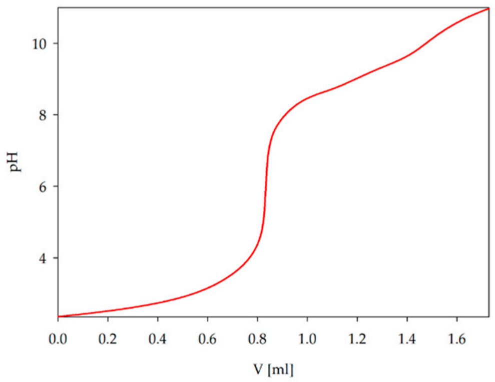

(a)

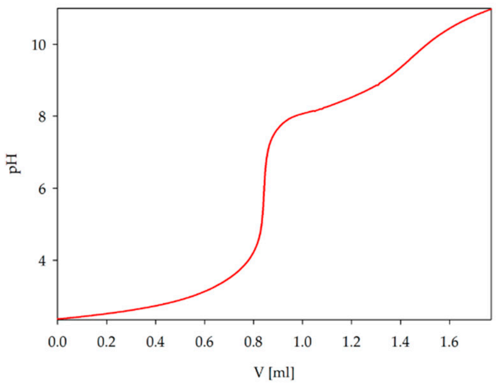

(b)

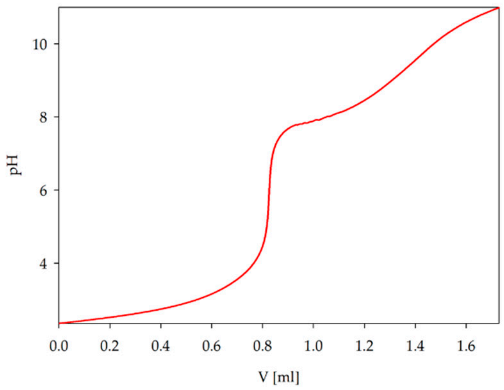

(c)

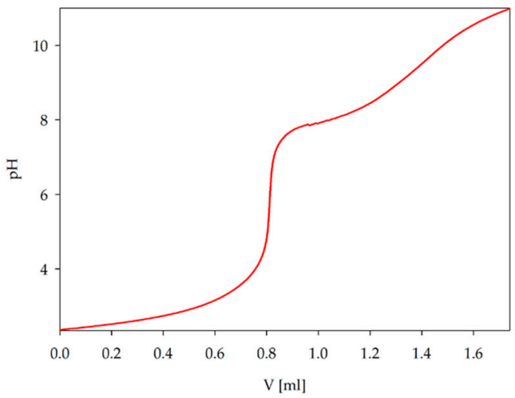

(d)

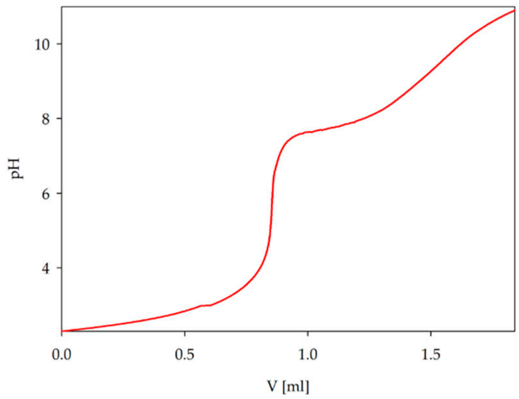

(e)

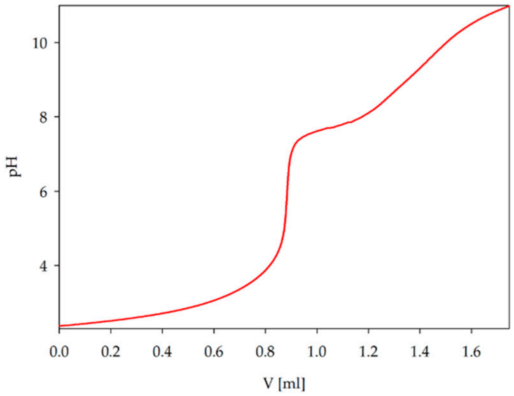

(f)

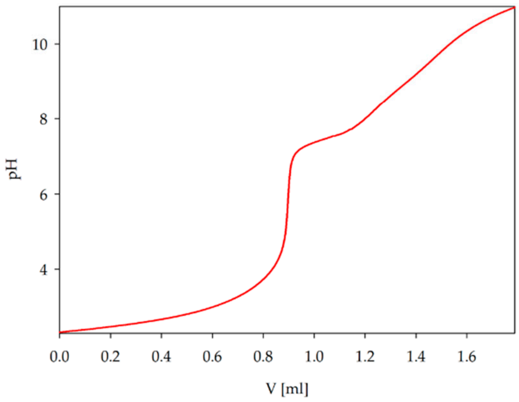

(g)

Figure S5. The titration curve for the systems: a) La(III)/GA/Spm; b) Nd(III)/GA/Spm; c) Eu(III)/GA/Spm; d) Tb(III)/GA/Spm; e) Ho(III)/GA/Spm; f) Lu(III)/GA/Spm.
